# The regulatory and synergistic effects of FBP2 and HKDC1 on glucose metabolism and malignant progression in gastric cancer

**DOI:** 10.1038/s41419-025-07997-z

**Published:** 2025-10-16

**Authors:** Zhiyuan Yu, Rui Li, Qixuan Xu, Chen Liang, Jingwang Gao, Zhen Yuan, Ruiyang Zhao, Wenquan Liang, Bo Cao, Xudong Zhao, Bo Wei, Peiyu Li

**Affiliations:** 1 School of Medicine, Nankai University, Tianjin, China; 2 Medical School of Chinese PLA, Beijing, China; 3 Department of General Surgery, The First Medical Center, Chinese PLA General Hospital, Beijing, China; 4 Department of Gastroenterology, Beijing Jishuitan Hospital, Capital Medical University, Beijing, China; 5 Department of Digestive Surgery, Linfen Central Hospital, Linfen, Shanxi China

**Keywords:** Gastric cancer, Biomarkers

## Abstract

Fructose-1,6-bisphosphatase (FBPase) serves as the rate-limiting enzyme in gluconeogenesis and can be categorized into two subtypes: FBP1 and FBP2. FBP1 has been reported to exhibit reduced expression and impaired function in various malignant tumors. However, there is limited research investigating the role of FBP2 in tumorigenesis. Our results showed that the expression level of FBP2 in gastric cancer (GC) tissues was reduced compared to that in adjacent non-tumor tissues. Low FBP2 expression was correlated with adverse clinicopathological characteristics and unfavorable prognosis. Overexpression of FBP2 in GC cells resulted in a decreased expression level of hypoxia inducible factor-1α (HIF-1α), enhanced oxidative phosphorylation, and a modest reduction in glycolytic activity. Notably, the FBP2 has been shown to elevate the expression level of hexokinase domain-containing protein-1 (HKDC1). Both cellular and animal studies demonstrated that the overexpression of FBP2 or the knockdown of HKDC1 could attenuate the malignant biological behavior of GC. Moreover, the synergistic effect of these two approaches exerted a more potent anti-tumor response. Overall, the synergistic effect of FBP2 and HKDC1 can suppress the progression of GC through the promotion of oxidative phosphorylation and inhibition of glycolysis. FBP2 and HKDC1 are anticipated to serve as novel molecular markers for the diagnosis, targeted therapy, and prognosis of GC.

## Introduction

Gastric cancer (GC) ranks as one of the most prevalent malignant neoplasms globally. According to the most recent oncological statistics, it is the fifth most frequently diagnosed cancer and the fourth leading cause of cancer-related mortality among all malignancies [1, 2]. Owing to its high malignancy, rapid progression, the absence of specific diagnostic markers, and inadequate emphasis on early screening and diagnosis, a significant proportion of GC patients are diagnosed at an advanced stage, thereby losing the opportunity for radical surgical intervention [2, 3]. The molecular mechanisms underlying the occurrence and progression of GC are intricate, and current biomarkers and targeted therapies have not substantially improved diagnostic accuracy or patient prognosis. Therefore, investigating the pathogenesis of GC and identifying novel diagnostic and therapeutic targets holds significant clinical potential [4, 5].

The metabolic processes of normal and tumor cells exhibit marked differences. Tumor cells can modulate the synthesis and degradation of metabolites through multiple mechanisms, thereby providing the necessary materials and energy for their malignant proliferation and rapid growth. This phenomenon is referred to as tumor metabolic reprogramming [6, 7]. As the predominant metabolic reprogramming process, aerobic glycolysis represents a hallmark of tumorigenesis and significantly influences tumor progression. Cancer cells exhibit heightened metabolic activity. Even in the presence of adequate oxygen, they predominantly rely on glycolysis for energy production, resulting in substantial lactic acid accumulation and limited ATP generation. This phenomenon is known as the Warburg effect, which is intricately linked to the generation of numerous tumor markers [7, 8].

Fructose-1,6-bisphosphatase (FBPase) is an enzyme that plays a crucial role in carbohydrate metabolism, specifically as the rate-limiting enzyme in gluconeogenesis. It catalyzes the hydrolysis of fructose-1,6-diphosphate into fructose-6-phosphate and inorganic phosphate. The loss of FBPase expression and function can result in the impairment or complete inhibition of gluconeogenesis, which indirectly facilitates the progression of glycolysis. FBPase can be classified into two subtypes, FBP1 and FBP2, which exhibit 77% structural homology. FBP1 and FBP2 are predominantly expressed in hepatic and muscular cells, respectively [9, 10]. Previous studies have demonstrated that FBP1 is correlated with the loss of expression and function in various malignant tumors, including liver cancer, lung cancer, and renal clear cell carcinoma. However, limited research has been conducted on the relationship between FBP2 and tumorigenesis [11, 12]. Li’s study demonstrated that reduced expression of FBP2 was associated with an increased incidence of GC, and that the expression levels were significantly correlated with survival outcomes. FBP2 could inhibit glucose metabolism and induce tumor cell apoptosis through suppression of the Akt-mTOR signaling pathway. These findings indicated that the restoration of FBP2 expression holds potential as a promising targeted therapeutic strategy for GC [13]. Our bioinformatics analysis revealed that the expression of FBP2 was consistently downregulated in GC tissues. Consequently, we designed this study to investigate the impact of FBP2 on the malignant biological behavior of GC and elucidate its underlying regulatory mechanisms.

## Materials and methods

### Bioinformatics analysis

Microarray datasets including GSE13911, GSE19826, GSE79973, GSE118916, GSE26899, and GSE103236 were obtained from the Gene Expression Omnibus (GEO) (http://www.ncbi.nlm.nih.gov/geo/). The GSE13911, GSE19826, and GSE79973 datasets, which are based on the GPL570 platform, were normalized and integrated using the COMBAT method via R (version 2.15.3) software. The differential expression of genes was analyzed utilizing the limma R software package. By comparing the microarray data between the tumor and normal groups, three distinct sets of differentially expressed genes (DEGs) were identified. Thereafter, the three sets of DEGs were cross-analyzed to identify the common DEGs. Furthermore, the top 100 DEGs among the three groups were intersected to identify a comprehensive set of significant DEGs. The protein interaction network was constructed utilizing the STRING database (https://string-db.org) to validate protein interactions and to identify potential interacting proteins. Furthermore, the GEPIA online platform (http://gepia.cancer-pku.cn/) was utilized for comprehensive pan-cancer analysis and validation of protein interactions.

### Clinical tissue specimens and microarrays

Clinical tissue specimens were obtained from 20 patients diagnosed with gastric adenocarcinoma who underwent radical surgery at the Chinese People’s Liberation Army (PLA) General Hospital between 2023 and 2024. Tissue microarrays were obtained from 95 patients admitted between 2016 and 2017. Paracancerous tissue specimens were obtained from normal tissue located at least 5 cm away from the tumor lesion margin. Furthermore, the clinicopathological data of 95 patients included in the tissue microarray study were systematically collected. Patients underwent radical surgery and were followed up for a period of 3–5 years post-surgery, with survival time calculated from the date of the surgical procedure. None of the patients enrolled in this study underwent neoadjuvant therapy, and all pathological diagnoses were verified by a minimum of two senior pathologists.

### Immunohistochemical (IHC) staining

GC tissues were subjected to fixation, embedding, sectioning, and staining. IHC staining was conducted utilizing the Metal-Enhanced DAB Substrate Kit (Solarbio, China) in strict adherence to the manufacturer’s protocol. Staining intensity was scored on a scale of 0–3, corresponding to negative, weak, moderate, and strong staining intensities, respectively. Staining extent was evaluated on a scale of 0 to 4, representing the percentage of positive cells as follows: 0%–5%, 6%–25%, 26%–50%, 51%–75%, and >75%. The IHC scores were independently assessed by two expert pathologists under double-blind conditions, and the final IHC score was calculated by multiplying the staining intensity score by the staining extent score. A final score of >6 was considered positive expression, and that of ≤6 was deemed negative expression.

### Cell culture

The human GC cell lines MKN-28, MGC-803, BGC-823, SGC-7901, and AGS utilized in this study are adherent cell lines procured from the Shanghai Cell Bank of the Chinese Academy of Sciences. Cell culture was conducted using a high-glucose complete medium supplemented with 10% fetal bovine serum (FBS) (Vazyme, China) and 1% penicillin-streptomycin-amphotericin solution (Servicebio, China). All cells were maintained in a humidified incubator at 37 °C with 5% CO_2_.

### Cell transfection

The overexpression, sh-RNA, and negative control plasmids utilized for cell transfection were designed and synthesized by Genomeditech (Shanghai, China) and Likoli Biotechnology (Beijing, China). The specifically designed lentiviral vector and its corresponding packaging plasmid were co-transfected into 293T cells using the HG transgene reagent. This process yielded lentiviruses capable of stably interfering with the expression of FBP2 and HKDC1, and their titers were subsequently determined. Subsequently, BGC-823 and AGS GC cell lines were transfected with the packaged lentivirus to establish stable cell lines characterized by FBP2 overexpression (FBP2) and HKDC1 knockdown (sh-HKDC1). The nucleotide sequences and plasmids used in this study are provided in Supplementary Material 1.

### Quantitative real-time PCR (qRT-PCR)

Total RNA was extracted from the cells utilizing the FastPure Cell/Tissue Total RNA Isolation Kit V2 (Vazyme, China) in strict accordance with the manufacturer’s protocol. The total RNA extracted was reverse-transcribed into cDNA utilizing the HiScript III All-in-one RT SuperMix for qPCR (Vazyme, China), and the subsequent PCR reaction was conducted using the Taq Pro Universal SYBR qPCR Master Mix (Vazyme, China). A 20 µL reaction mixture was prepared using 10 µL Taq Pro Universal SYBR qPCR Master Mix, 0.4 µL forward primer (10 µM), 0.4 µL reverse primer (10 µM), template cDNA, and ddH_2_O. The mixture was thoroughly combined and subjected to amplification on ABI7500 (Applied Biosystems, USA). GAPDH was utilized as the internal control, and the relative expression levels of target genes were quantified using the 2^−ΔΔct^ method. The primers utilized in this study were synthesized by Biomed (Beijing, China), and the primer sequences are provided in Supplementary Material 2.

### Western blot (WB)

Cell and tissue proteins were extracted using RIPA lysis buffer containing 1% PMSF. Following protein quantification by the BCA method (Beyotime, China), the proteins were separated by SDS-polyacrylamide gel electrophoresis and subsequently transferred onto a polyvinylidene fluoride membrane. The membrane was blocked with 5% skim milk at room temperature for 2 h to prevent nonspecific binding. The membrane was subsequently incubated with the primary antibody overnight at 4 °C, followed by incubation with an HRP-conjugated secondary antibody for 1 h at room temperature. The protein bands were visualized using an ECL chemiluminescence solution (Beyotime, China). The antibodies utilized in this study are listed in Supplementary Material 3.

### Cell Counting Kit-8 (CCK-8) assay

A total of 3000 GC cells were seeded into each well of the 96-well plates. Following cell adhesion, 10 µL CCK-8 reagent (Vazyme, China) was added to each well at 24, 48, 72, and 96 h, respectively. The plates were then incubated in a dark environment at 37 °C for 1 h. Finally, the 96-well plates were placed into an enzyme-labeled instrument, and the absorbance of each well was measured at 450 nm.

### Colony formation assay

GC cells in optimal growth conditions and during the logarithmic growth phase were resuspended and inoculated into six-well plates at a density of 700 cells per well. Following continuous culture for 10 days, the cells were fixed with 4% paraformaldehyde, stained with crystal violet, and subsequently photographed and counted.

### Cell scratch assay

GC cells in optimal growth conditions and during the logarithmic growth phase were resuspended and inoculated into six-well plates. When the cell density in the 6-well plate reaches 80%–90%, a 200 μL pipette tip is used to create uniform and vertical scratches. Discard the old medium and wash the cells with PBS buffer. Subsequently, add 2 mL FBS-free high-glucose medium, then observe the scratched area under a microscope and record the initial cell status at 0 h. Following incubation periods of 12 and 24 h in the incubator, microscopic images were captured. The scratch widths at these respective time points were subsequently quantified using image analysis software.

### Transwell invasion assay

The matrix glue was evenly applied to the bottom of the Transwell chamber (CORNING, USA) and incubated to allow for solidification. The nutrient-deprived cells were cultured until they reached the logarithmic growth phase. Subsequently, the cells were trypsinized, resuspended, and counted, and the cell concentration was then adjusted to 5 × 10^5^ cells/mL. 750 μL 20% complete medium was added to the lower chamber of the Transwell insert, followed by the addition of 100 μL diluted cell suspension into the upper chamber. The assembly was then incubated for 24 h under standard conditions. Following the removal of the media, the cells adhering to the underside of the chamber were fixed using 4% paraformaldehyde, stained with crystal violet, and subsequently imaged and quantified.

### Glycolysis

Lactate, pyruvate, and ATP production, glucose uptake rates, and Seahorse glycolysis stress tests were utilized to evaluate cellular glycolytic activity. The Lactate Assay Kit, Pyruvate Assay Kit, ATP Assay Kit (Solarbio, China), Glucose Uptake Colorimetric Assay Kit (Biovision, USA), and Seahorse XF glycolysis stress test kit (Agilent, USA) were utilized to measure the aforementioned indicators in accordance with the respective manufacturer’s protocols. Absorbance is quantified using an enzyme-labeled method at the respective optimal wavelength.

### Xenograft tumors in mice

Four-week-old male BALB/C nude mice were purchased from Sipeifu Biotechnology Co., Ltd. (Beijing, China). Prior to the commencement of the formal experiment, the mice were housed in a specific pathogen-free (SPF) environment for 1 week. During the acclimatization period, animals were provided with purified water and sterile feed. Aseptic bedding was replaced every three days. The ambient temperature was maintained at 20 °C–26 °C, while the relative humidity was kept at 50%–60%. A light/dark cycle of 12 h each is also maintained. Subsequently, the mice were randomly allocated into four groups: FBP2+sh-HKDC1, FBP2+sh-NC, sh-HKDC1+Vector, and Vector+sh-NC. The grouping of numbered nude mice was performed according to the random number table method. To ensure the credibility and validity of the comparison results, a minimum of five nude mice were included in each group. 5 × 10^6^ treated BGC-823 GC cells were inoculated subcutaneously into the right dorsal region of the mice. The mice were continuously fed according to the aforementioned protocol, and subcutaneous tumor volume was assessed every three days. Following a 28-day period, the animals underwent in vivo imaging analysis, after which the tumors were excised for volumetric measurement and lactic acid content determination. In animal experiments, blinding was employed exclusively for researchers during the evaluation of results.

### Statistical analysis

All experiments were conducted in triplicate. Statistical analyses and graphical representations were conducted utilizing SPSS 26.0, R 2.15.3, and GraphPad Prism 10.0 software. For continuous variables with normal distribution, data were presented as mean ± standard deviation (SD), and inter-group comparisons were performed using the unpaired, two-tailed Student’s t-test. For continuous variables with non-normal distribution, data were presented as median (interquartile range), and inter-group comparisons were performed using the Mann–Whitney U test. For categorical variables, frequency distributions and proportions were utilized for representation, while inter-group comparisons were conducted using Chi-square or Fisher’s exact tests as appropriate. The Kaplan–Meier survival curves were plotted to conduct survival analysis, and the log-rank test was employed to perform pairwise comparisons between groups. The quantitative analysis of the WB data was performed using the ImageJ software. The gray value of the target protein was normalized by dividing it by the gray value of the internal reference protein (β-actin). Subsequently, the control group was designated as 1, and the relative expression level of the target protein in the experimental group was calculated. *P* < 0.05 was considered statistically significant.

## Results

### Bioinformatics database analysis

**Fig. 1:**
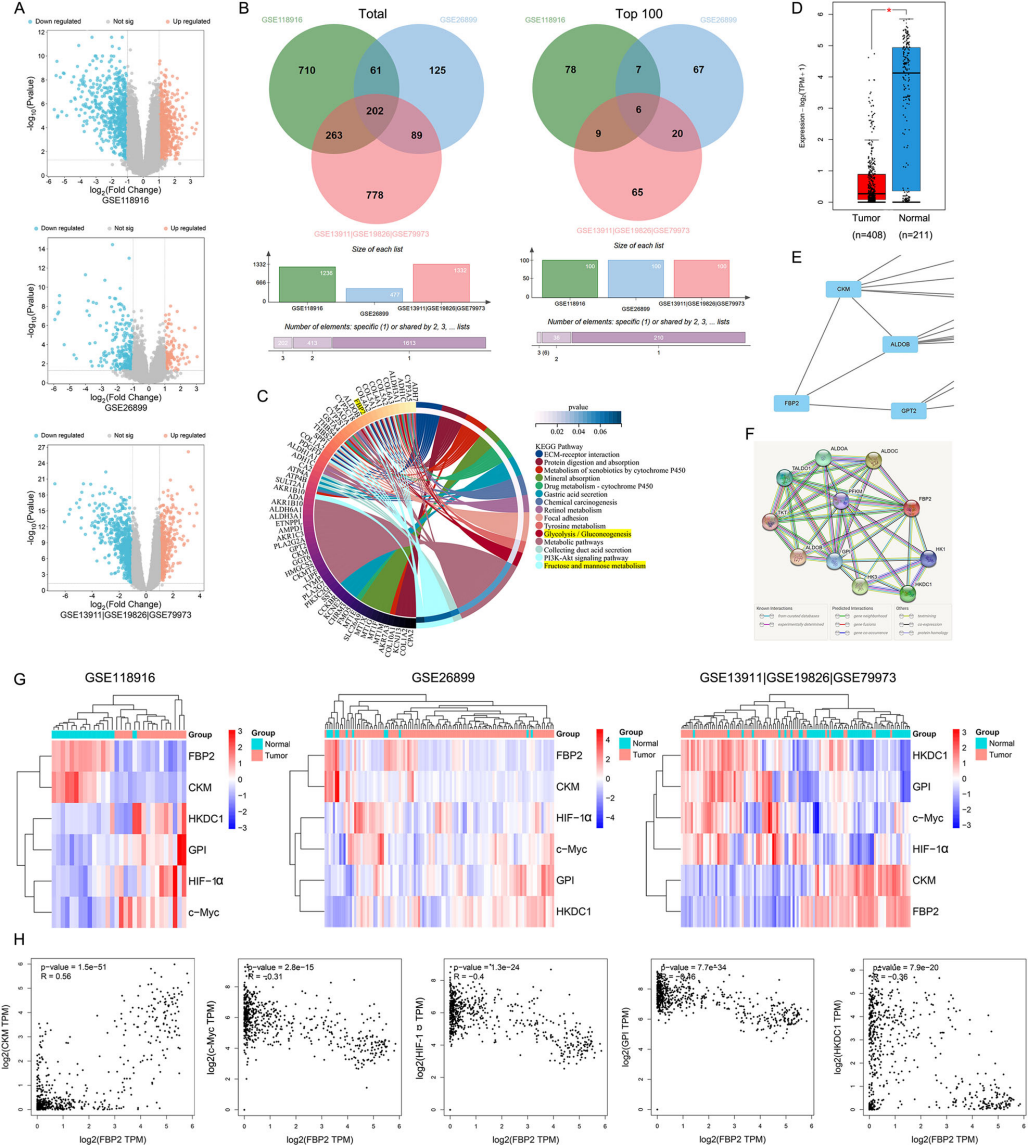
Comprehensive bioinformatics analysis of key genes involved in GC. **A** Volcanic plots of DEGs based on multiple GC microarray datasets from the GEO database. **B** Venn diagrams based on the total and the top 100 most significant DEGs from three microarray datasets. **C** KEGG enrichment conducted using the 202 DEGs. **D** Evaluation of FBP2 expression levels in GC versus adjacent tissues utilizing the TCGA and GTEx databases. **E** Proteins that interact with FBP2 within the protein interaction network established based on the aforementioned DEGs. **F** Prediction of FBP2-interacting proteins utilizing the STRING database. **G** Heatmaps illustrating the gene expression profiles of FBP2 and its interacting proteins derived from three distinct microarray datasets. **H** Correlation analysis of FBP2 with CKM, c-Myc, HIF-1α, GPI, and HKDC1.

Following a rigorous screening process, we retrieved and downloaded five gene datasets (GSE13911, GSE19826, GSE79973, GSE118916, and GSE26899) from the GEO database. These datasets encompassed microarray data from 171 GC and 80 adjacent non-tumor tissues (Table 1). Subsequently, we integrated three datasets from the GPL570 platform to eliminate batch effects and generate a consolidated dataset (GSE13911|GSE19826|GSE79973). Through the comprehensive analysis of three distinct datasets, we identified three sets of DEGs in GC tissues compared to adjacent non-tumor tissues (Fig. 1A). By analyzing the intersection of the three groups of DEGs, a total of 202 DEGs were identified, among which 6 (FBP2, KCNE2, CHGA, TRIM50, ESRRG, and ADH7) exhibited significant differences (Fig. 1B). KEGG enrichment analysis conducted using 202 DEGs indicated that several DEGs, such as FBP2, were enriched in pathways related to glucose metabolism (Fig. 1C).

**Table 1:** Characteristics of included GEO datasets.

Datasets	Platforms	Region	Experimental (Sample size)	Control (Sample size)	Submission date (year)
GSE13911	GPL570	Italy	Tumor (38)	Normal (31)	2008
GSE19826	GPL570	China	Tumor (12)	Normal (12)	2010
GSE79973	GPL570	China	Tumor (10)	Normal (10)	2016
GSE118916	GPL15207	China	Tumor (15)	Normal (15)	2019
GSE26899	GPL6947	USA	Tumor (96)	Normal (12)	2016

*GEO* Gene Expression Omnibus, *GSE* GEO datasets, *GPL* GEO platforms.

FBP2 was selected for further investigation. Analysis conducted utilizing the TCGA and GTEx databases also revealed that FBP2 exhibited significant differential expression (Fig. 1D). Pan-cancer analysis revealed that FBP2 expression is significantly downregulated in GC and testicular cancer, with particularly low expression observed in GC. Previous studies have demonstrated that HIF-1α and c-Myc interact with FBP2. Subsequently, protein-protein interaction (PPI) networks for the 202 DEGs were constructed using the STRING database. This analysis yielded three protein-coding genes that are directly associated with FBP2 (Fig. 1E). Simultaneously, an additional ten protein-coding genes potentially associated with FBP2 were identified using the STRING database (Fig. 1F). The analysis of the aforementioned 15 protein-coding genes in the bioinformatics database revealed that only CKM, c-Myc, HIF-1α, GPI, and HKDC1 showed differential expression (Fig. 1G) and exhibited a correlation with FBP2 (Fig. 1H).

### FBP2 is lowly expressed in GC tissues

**Fig. 2:**
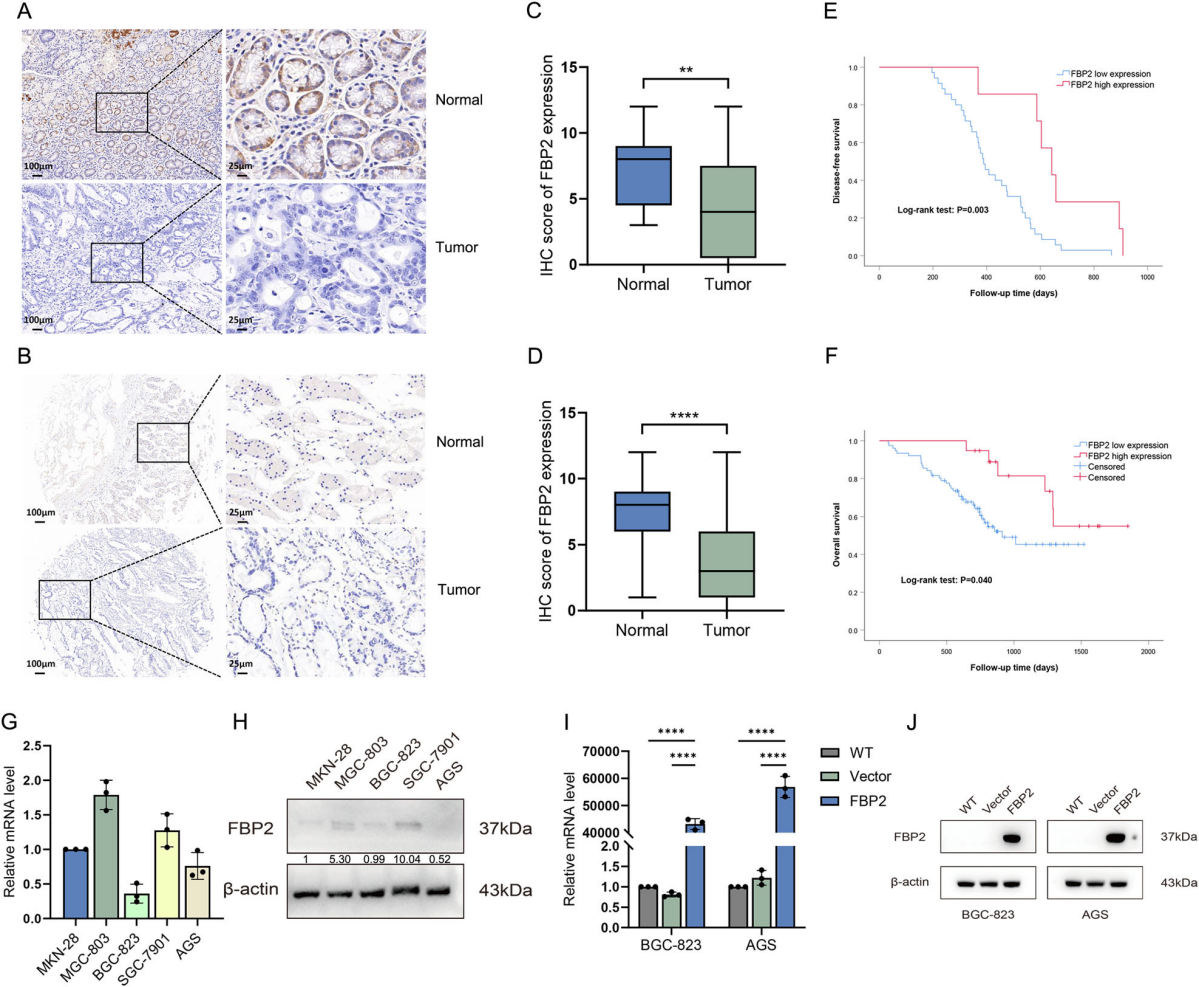
The expression and clinical significance of FBP2 in GC tissues, as well as the establishment and validation of FBP2 overexpression in GC cell lines. IHC staining of FBP2 using **A** newly collected tissue specimens and **B** previously established tissue microarrays. IHC scores derived from **C** 20 paired tissue samples and **D** 95 paired tissue microarrays. **E** Overall survival (OS) and **F** disease-free survival (DFS) analyses both demonstrated a significant improvement in survival rates among patients with FBP2-positive GC. **G** qRT-PCR and **H** WB were employed to assess the expression levels of FBP2 in five GC cell lines. **I** qRT-PCR and **J** WB analysis confirmed the successful establishment of the FBP2 overexpression cell line. **P < 0.01, ****P < 0.0001.

We acquired fresh tissue specimens from 20 patients diagnosed with gastric adenocarcinoma and tissue microarrays from an additional 95 patients with the same diagnosis. IHC staining was conducted on both tissue specimens (Fig. 2A) and tissue microarrays (Fig. 2B). IHC results revealed that the positive expression rates of FBP2 in GC and adjacent non-tumor tissues were 20.9% (24/115) and 64.3% (74/115), respectively. A statistically significant difference was observed between these two groups (χ^2^ = 44.450, *P* < 0.001). Furthermore, the IHC staining scores for FBP2 in GC tissues were 3 (1, 6), whereas those in adjacent non-tumor tissues were 8 (6, 9). The difference in IHC staining scores between the two groups was statistically significant, irrespective of whether the samples were tissue specimens (Z = −2.707, *P* = 0.007) (Fig. 2C) or tissue microarrays (Z = −6.497, *P* < 0.001) (Fig. 2D).

### FBP2 expression is associated with clinicopathological characteristics and prognosis

Based on the IHC scoring of GC tissue microarrays, 95 patients were categorized into positive and negative groups. The results demonstrated statistically significant differences between the two groups in terms of T stage (*P* = 0.020), N stage (*P* = 0.028), pathological TNM stage (*P* = 0.033), histological types (*P* = 0.007), and presence of lymphovascular invasion (*P* = 0.012) (Table 2). To evaluate the impact of FBP2 expression on survival outcomes, we conducted a log-rank test to compare disease-free survival (DFS) and overall survival (OS) between the FBP2-positive and FBP2-negative groups. The results demonstrated that patients with FBP2-positive expression had significantly better DFS (*P* = 0.003) (Fig. 2E) and OS (*P* = 0.040) (Fig. 2F).

**Table 2:** Correlation analysis of FBP2 expression levels and clinicopathological characteristics in gastric cancer.

Clinicopathological characteristics	Number	FBP2 expression levels		χ^2^	*P*-value
		Positive, n (%)	Negative, n (%)		
Age				1.579	0.209
≤60 years	38	10 (52.6)	28 (36.8)		
>60 years	57	9 (47.4)	48 (63.2)		
Sex				0.589	0.443
Male	71	16 (84.2)	55 (72.4)		
Female	24	3 (15.8)	21 (27.6)		
Tumor size				3.821	0.051
≤5 cm	51	14 (73.7)	37 (48.7)		
>5 cm	44	5 (26.3)	39 (51.3)		
T stage				5.429	**0.020**
T1-2	25	9 (47.4)	16 (21.1)		
T3-4	70	10 (52.6)	60 (78.9)		
N stage				4.849	**0.028**
N0	20	8 (42.1)	12 (15.8)		
N1-3	75	11 (57.9)	64 (84.2)		
Tumor stage (pathological)				7.00	**0.033**
I–II	35	11 (57.9)	24 (31.6)		
III–IV	60	8 (42.1)	52 (68.4)		
Histological types				7.168	**0.007**
Differentiated	14	7 (36.8)	7 (9.2)		
Undifferentiated	81	12 (63.2)	69 (90.8)		
Lymphovascular invasion				6.264	**0.012**
Absence	56	16 (84.2)	40 (52.6)		
Presence	39	3 (15.8)	36 (47.4)		
Nerve invasion				0.810	0.368
Absence	67	15 (78.9)	52 (68.4)		
Presence	28	4 (21.1)	24 (31.6)		

The bold values indicate statistical significance (*P* < 0.05).

### Construction of FBP2 cell lines

qRT-PCR (Fig. 2G) and WB assays (Fig. 2H) revealed that the expression level of FBP2 was decreased in all tested GC cell lines, with particularly low expression observed in BGC-823 and AGS cells. Consequently, the BGC-823 and AGS cell lines were selected for subsequent experiments. The transfection efficiency of the lentivirus was validated using qRT-PCR (Fig. 2I) and WB assays (Fig. 2J), thereby confirming the successful establishment of the FBP2 cell lines. In the subsequent description, the “FBP2 cells” refer to cell lines that overexpress FBP2.

### FBP2 inhibits the malignant biological behavior of GC cells

**Fig. 3:**
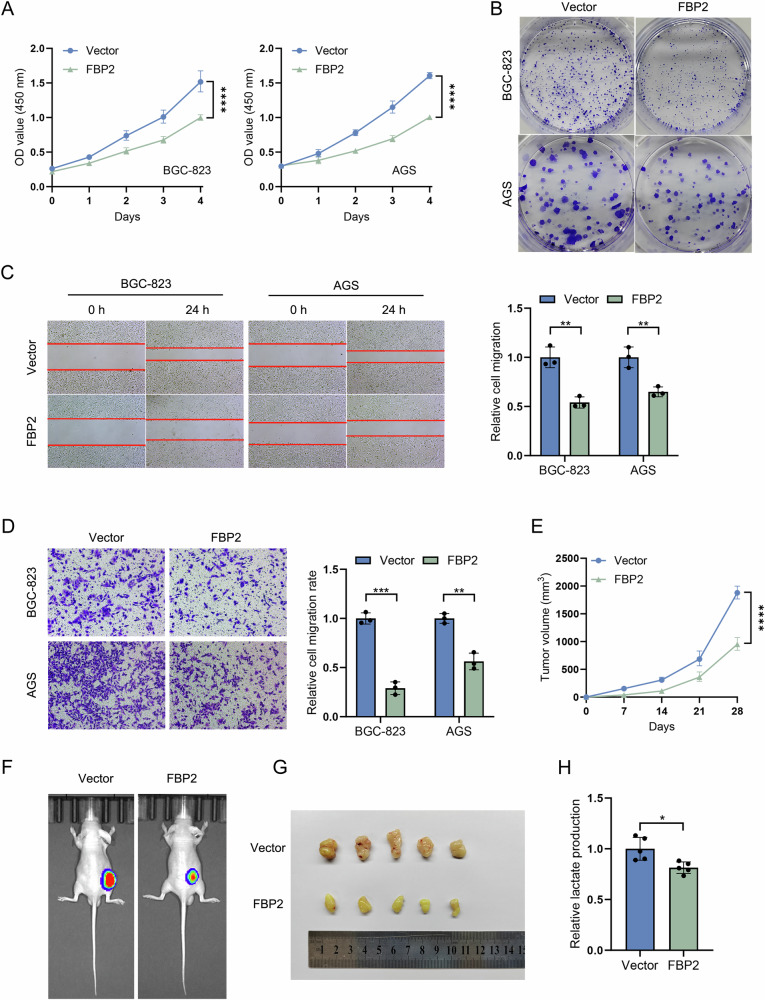
Inhibition of malignant biological behaviors of GC cells by FBP2 in vitro and in vivo. **A** CCK-8 assays and **B** colony formation assays confirmed the inhibitory effect of FBP2 on the proliferation of BGC-823 and AGS GC cell lines. **C** Scratch assays and **D** Transwell assays confirmed the inhibitory effects of FBP2 on the migratory and invasive capabilities of BGC-823 and AGS GC cell lines. The bar chart presented the statistical analysis of the migration and invasion ratios. **E** Trend of subcutaneous tumor volume in nude mice following the injection of BGC-823 GC cells over a four-week period. **F** In vivo imaging, **G** tumor excision, and **H** lactic acid production were evaluated in nude mice from the FBP2 and Vector groups four weeks post-subcutaneous injection. “FBP2 cells” refers to cell lines that overexpress FBP2. *P < 0.05, **P < 0.01, ***P < 0.001, ****P < 0.0001.

The results of CCK-8 (Fig. 3A) and colony formation assays (Fig. 3B) demonstrated that the proliferative capacity of FBP2 cell lines was markedly diminished. The results of the cell scratch (Fig. 3C) and Transwell assays (Fig. 3D) demonstrated that FBP2 significantly suppressed the migratory and invasive capabilities of GC cells. After establishing the effects of FBP2 through in vitro experiments, we subsequently confirmed these findings through in vivo experiments. We established subcutaneous tumor models in nude mice for both the FBP2 and Vector groups. Based on the tumor measurement data, we generated a line graph depicting the temporal changes in subcutaneous tumor volume. The results demonstrated that the subcutaneous tumor growth rate was reduced in the FBP2 group (Fig. 3E). Furthermore, in vivo imaging results demonstrated a significant reduction in the luminescence intensity of subcutaneous tumors in the FBP2 group (Fig. 3F). Following the euthanasia of nude mice and excision of subcutaneous tumors (Fig. 3G), analysis revealed that lactic acid production within the tumor was reduced in the FBP2 group (Fig. 3H).

### Inhibitory mechanism of FBP2 in oncogenesis

**Fig. 4:**
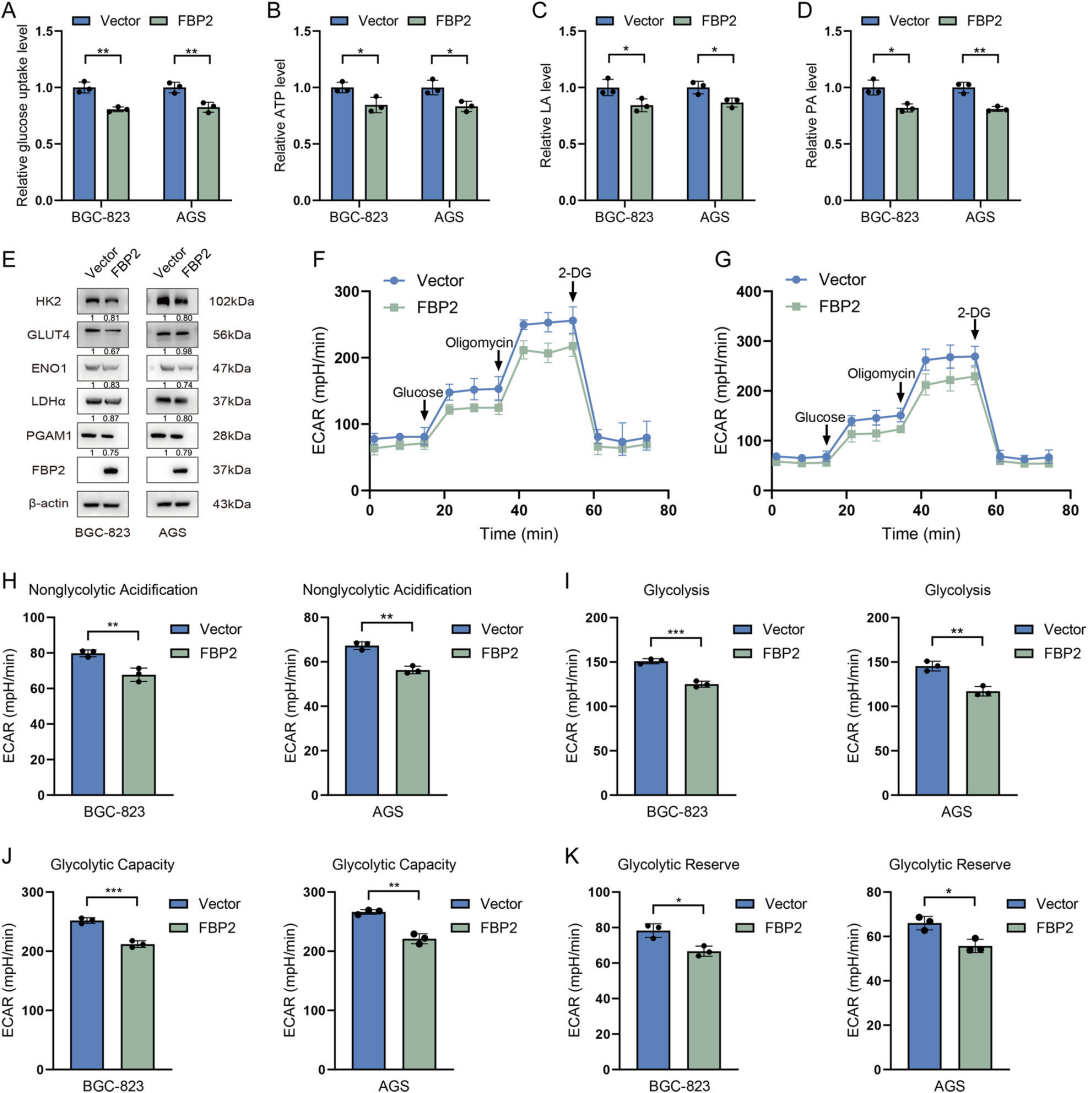
FBP2 has the capability to inhibit aerobic glycolysis in GC cells. **A** The glucose uptake rate, **B** ATP synthesis, **C** lactic acid (LA), **D** and pyruvate acid (PA) production of FBP2 and Vector cells were measured and compared using ultraviolet spectrophotometry. **E** Impact of FBP2 on the expression levels of key glycolytic proteins in GC cells. Line chart depicting the alterations in glycolytic pressure in **F** BGC-823 and **G** AGS GC cells with FBP2 overexpression. **H** Analysis of nonglycolytic acidification, **I** glycolysis, **J** glycolytic capacity, and **K** glycolytic reserve between FBP2 and Vector GC cells using extracellular acidification rate (ECAR) analysis. “FBP2 cells” refers to cell lines that overexpress FBP2. Data are presented as the mean ± standard deviation, n = 3. *P < 0.05, **P < 0.01, ***P < 0.001.

First, we examined the impact of FBP2 on the aerobic glycolysis capacity in GC cells. The glucose uptake rate (Fig. 4A), ATP production (Fig. 4B), as well as the generation of lactic acid (LA) (Fig. 4C) and pyruvate acid (PA) (Fig. 4D) were reduced in the FBP2 group. WB analysis revealed that the expression levels of five key glycolytic pathway proteins (PGAM1, LDHα, ENO1, GLUT4, and HK2) were reduced following FBP2 overexpression (Fig. 4E). Furthermore, to comprehensively assess the impact of FBP2 on metabolic activity, the alterations in the extracellular acid production rate (ECAR) of BGC-823 (Fig. 4F) and AGS (Fig. 4G) cells were investigated. Following the FBP2 overexpression, GC cells exhibited a reduction in nonglycolytic acidification (Fig. 4H), glycolysis (Fig. 4I), glycolytic capacity (Fig. 4J), and glycolytic reserve (Fig. 4K).

**Fig. 5:**
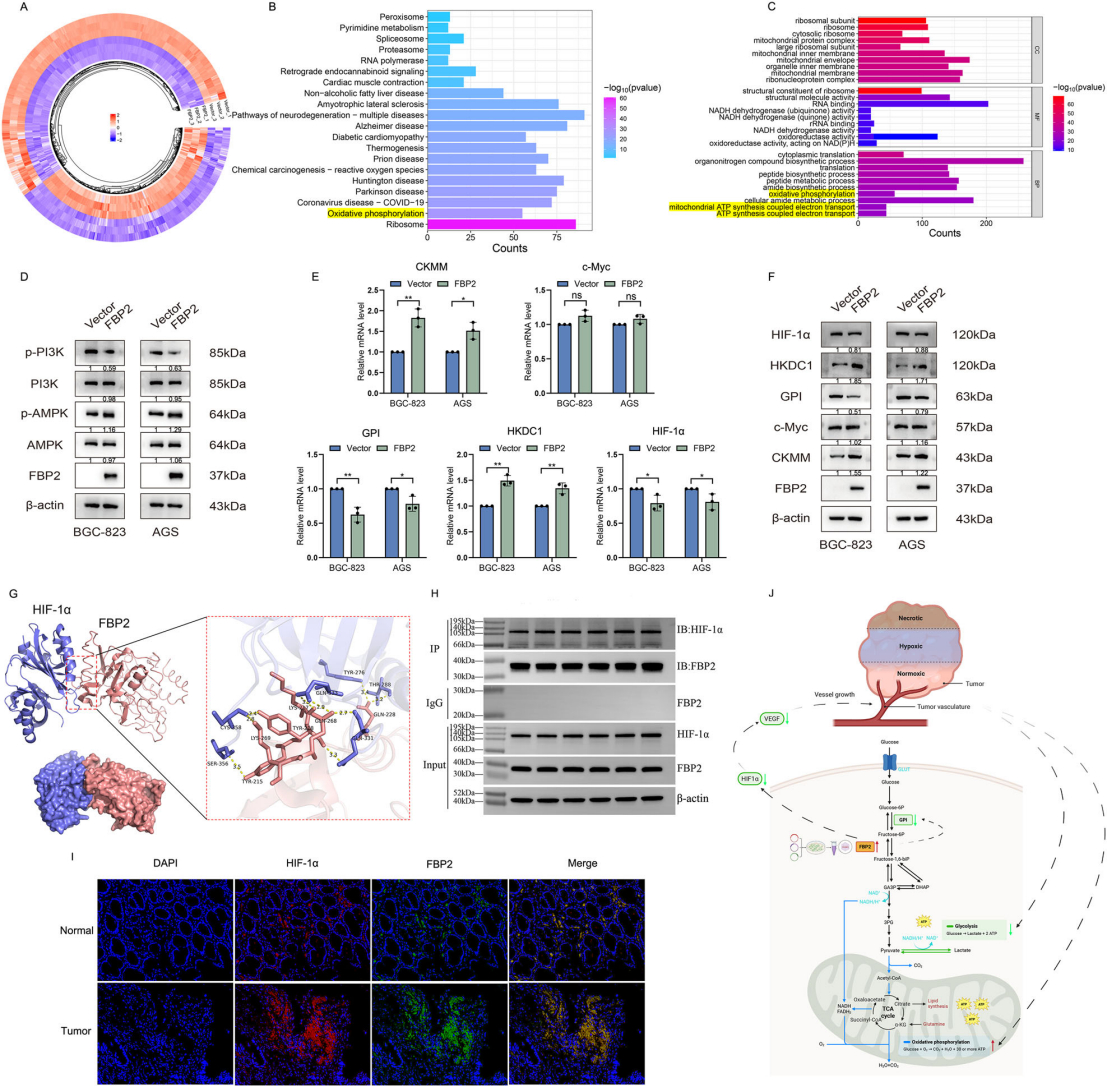
Impact of FBP2 on oxidative phosphorylation in GC cells and analysis of FBP2-interacting proteins. **A** Heatmap generated from the RNA sequencing data of FBP2 and Vector GC cells, along with **B** enrichment analysis of upregulated KEGG and **C** GO pathways. **D** Impact of FBP2 on the expression levels of key oxidative phosphorylation proteins in GC cells. **E** qRT-PCR and **F** WB assays were employed to assess the impact of FBP2 on the expression levels of interaction proteins in GC cells. **G** The optimal conformation and binding site of HIF-1α and FBP2 were predicted through molecular docking analysis. **H** The CO-IP assay utilizing six FBP2 GC cell samples was employed to investigate the authentic interaction status between FBP2 and HIF-1α. **I** Immunofluorescence heteroduplex staining of HIF-1α and FBP2 in GC and adjacent normal tissues. **J** Schematic representation of the regulatory mechanism of FBP2 in GC progression. “FBP2 cells” refers to cell lines that overexpress FBP2. *P < 0.05, **P < 0.01.

We collected FBP2 and Vector GC cells for RNA sequencing analysis, and identified DEGs with a Fold change ≥2 and *P* < 0.05 between the two groups. A heatmap (Fig. 5A) was constructed to visually represent the data. Following this, the enrichment analysis of KEGG (Fig. 5B) and GO pathways (Fig. 5C) based on the upregulated DEGs revealed significant enrichment in oxidative phosphorylation, redox reactions, and other metabolic pathways. WB analysis revealed a significant increase in the expression level of phospho-AMPK (p-AMPK), a crucial protein involved in oxidative phosphorylation, in the FBP2 group. Furthermore, FBP2 has been shown to decrease the expression level of phospho-PI3K (p-PI3K) protein (Fig. 5D).

qRT-PCR (Fig. 5E) and WB (Fig. 5F) analyses both revealed that FBP2 interacts with CKMM, HKDC1, GPI, and HIF-1α. Existing literature has established that HIF-1α can facilitate tumor progression through the induction of neovascularization and the amelioration of hypoxic conditions. Consequently, we conducted an extensive series of analyses to investigate the PPI between HIF-1α and FBP2. Utilizing molecular docking technology, we predicted the binding site of FBP2 and HIF-1α, identified the optimal conformation for visualization using PyMOL, and generated the docking structure diagram of FBP2 and HIF-1α. The amino acid residues of HIF-1α and FBP2 are capable of forming multiple stable hydrogen bonds, indicating that these two proteins can interact to form relatively stable complexes (Fig. 5G). The Co-Immunoprecipitation (CO-IP) assay conducted on FBP2 GC cells demonstrated a significant interaction between the FBP2 and HIF-1α protein (Fig. 5H). The immunofluorescence heterodouble labeling assays performed on patients’ tissue sections also demonstrated that FBP2 and HIF-1α colocalize in GC and adjacent tissues, suggesting a potential direct interaction between these two proteins (Fig. 5I). Based on the findings from the aforementioned experiments, we elucidated the mechanism by which FBP2 regulates GC (Fig. 5J).

### Synergistic regulation of FBP2 and HKDC1 on the malignant biological behavior of GC cells

**Fig. 6:**
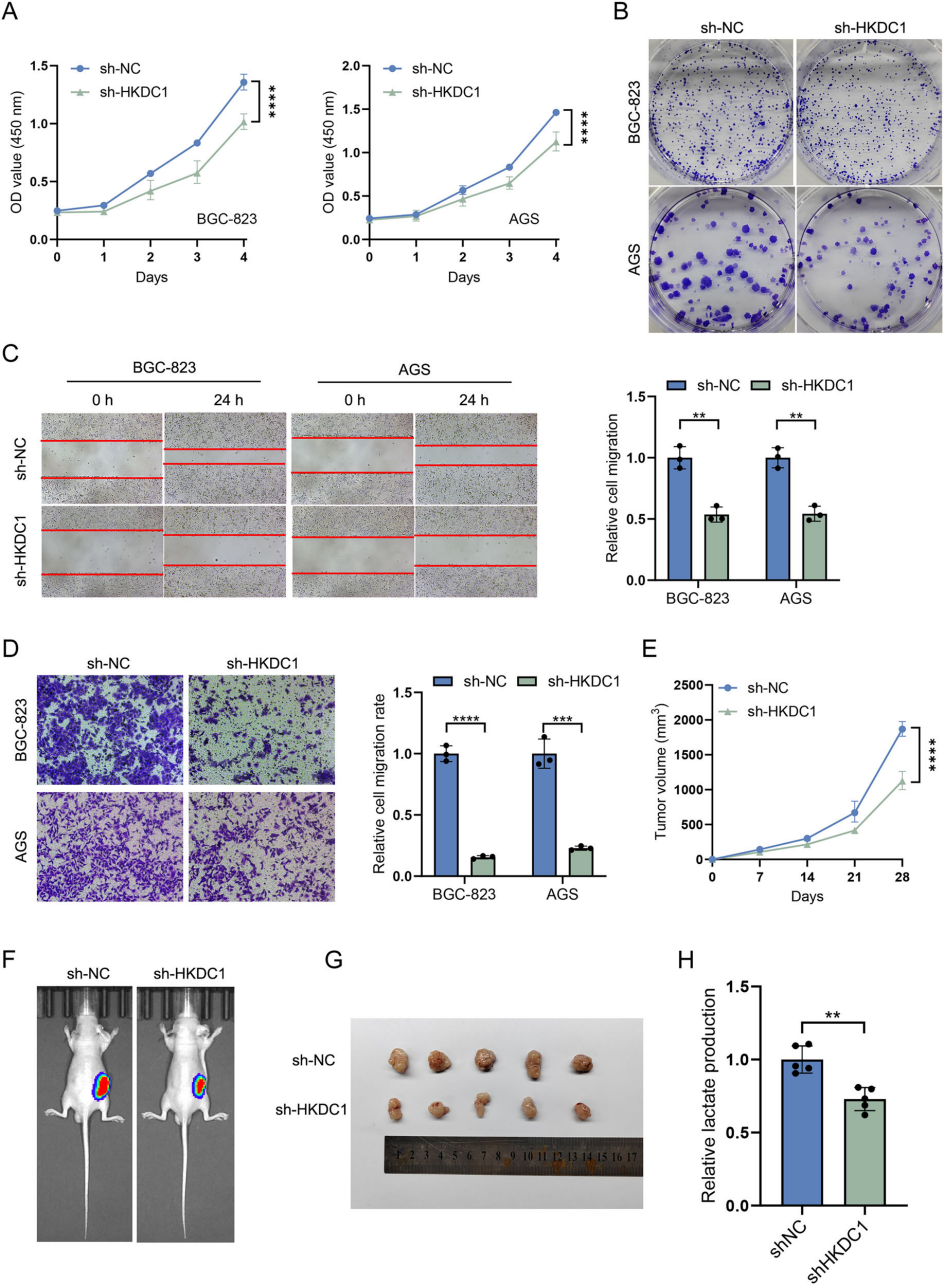
Inhibition of malignant biological behaviors of GC cells by sh-HKDC1 in vitro and in vivo. **A** CCK-8 assays and **B** colony formation assays confirmed the inhibitory effect of sh-HKDC1 on the proliferation of BGC-823 and AGS GC cell lines. **C** Scratch assays and **D** Transwell assays confirmed the inhibitory effects of sh-HKDC1 on the migratory and invasive capabilities of BGC-823 and AGS GC cell lines. The bar chart presented the statistical analysis of the migration and invasion ratios. **E** Trend of subcutaneous tumor volume in nude mice following the injection of BGC-823 GC cells over a four-week period. **F** In vivo imaging, **G** tumor excision, and **H** lactic acid production were evaluated in nude mice from the sh-HKDC1 and sh-NC groups four weeks post-subcutaneous injection. **P < 0.01, ***P < 0.001, ****P < 0.0001.

As the most recently identified member of the hexokinase family, HKDC1 is hypothesized to fulfill a comparable function to hexokinase in facilitating tumor progression through the regulation of glycolysis and inhibition of apoptosis. In the analysis of PPI, we observed that FBP2 resulted in an increased expression level of HKDC1. Consequently, we conducted an in-depth investigation into the synergistic effects of FBP2 and HKDC1. First, stable BGC-823 and AGS cell lines with reduced HKDC1 expression (sh-HKDC1) were established through lentiviral transfection. Subsequently, our in vitro experiments, including CCK-8 (Fig. 6A), colony formation (Fig. 6B), cell scratch (Fig. 6C), and Transwell assays (Fig. 6D), demonstrated that sh-HKDC1 significantly inhibited the proliferation, migration, and invasive capabilities of GC cells. In vivo experiments of subcutaneous tumor formation in nude mice demonstrated that the growth rate (Fig. 6E), luminescence intensity (Fig. 6F), tumor size (Fig. 6G), and lactic acid production (Fig. 6H) in the sh-HKDC1 group were reduced compared to the sh-NC group.

**Fig. 7:**
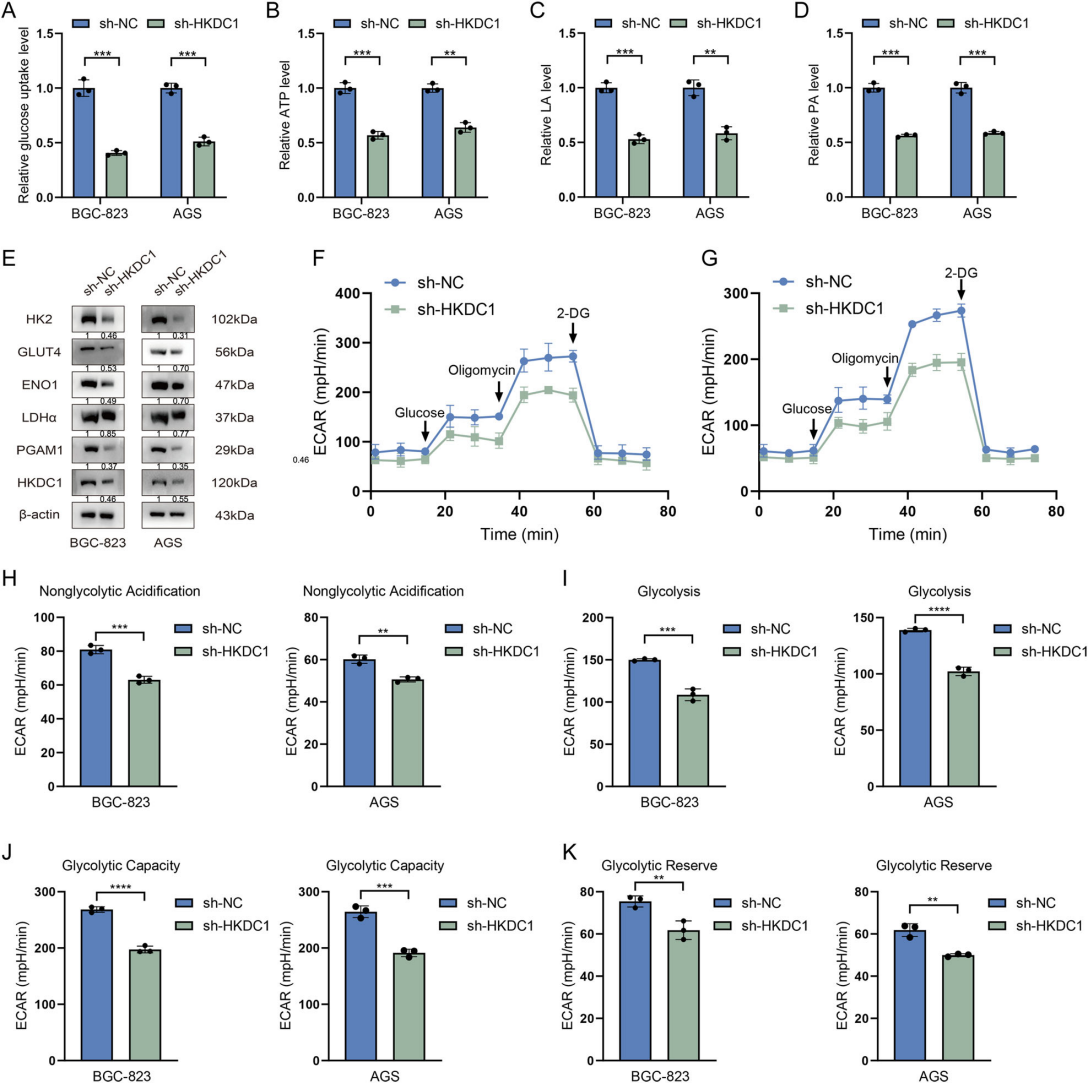
sh-HKDC1 has the capability to inhibit aerobic glycolysis in GC cells. **A** The glucose uptake rate, **B** ATP synthesis, **C** lactic acid (LA), and **D** pyruvate acid (PA) production of sh-HKDC1 and sh-NC cells were measured and compared using ultraviolet spectrophotometry. **E** Impact of HKDC1 on the expression levels of key glycolytic proteins in GC cells. Line chart depicting the alterations in glycolytic pressure in **F** BGC-823 and **G** AGS GC cells with sh-HKDC1. **H** Analysis of nonglycolytic acidification, **I** glycolysis, **J** glycolytic capacity, and **K** glycolytic reserve between sh-HKDC1 and sh-NC GC cells using extracellular acidification rate (ECAR) analysis. Data are presented as the mean ± standard deviation, n = 3. **P < 0.01, ***P < 0.001, ****P < 0.0001.

Subsequently, we examined the impact of sh-HKDC1 on the aerobic glycolysis capacity in GC cells. The glucose uptake rate (Fig. 7A), ATP production (Fig. 7B), as well as the generation of LA (Fig. 7C) and PA (Fig. 7D) were reduced in the sh-HKDC1 group. WB analysis revealed that the expression levels of key glycolytic pathway proteins were reduced following sh-HKDC1 (Fig. 7E). Furthermore, to comprehensively assess the impact of sh-HKDC1 on metabolic activity, the alterations in the ECAR of cells were investigated (Fig. 7F, G). Following the sh-HKDC1, GC cells exhibited a significant reduction in nonglycolytic acidification (Fig. 7H), glycolysis (Fig. 7I), glycolytic capacity (Fig. 7J), and glycolytic reserve (Fig. 7K).

**Fig. 8:**
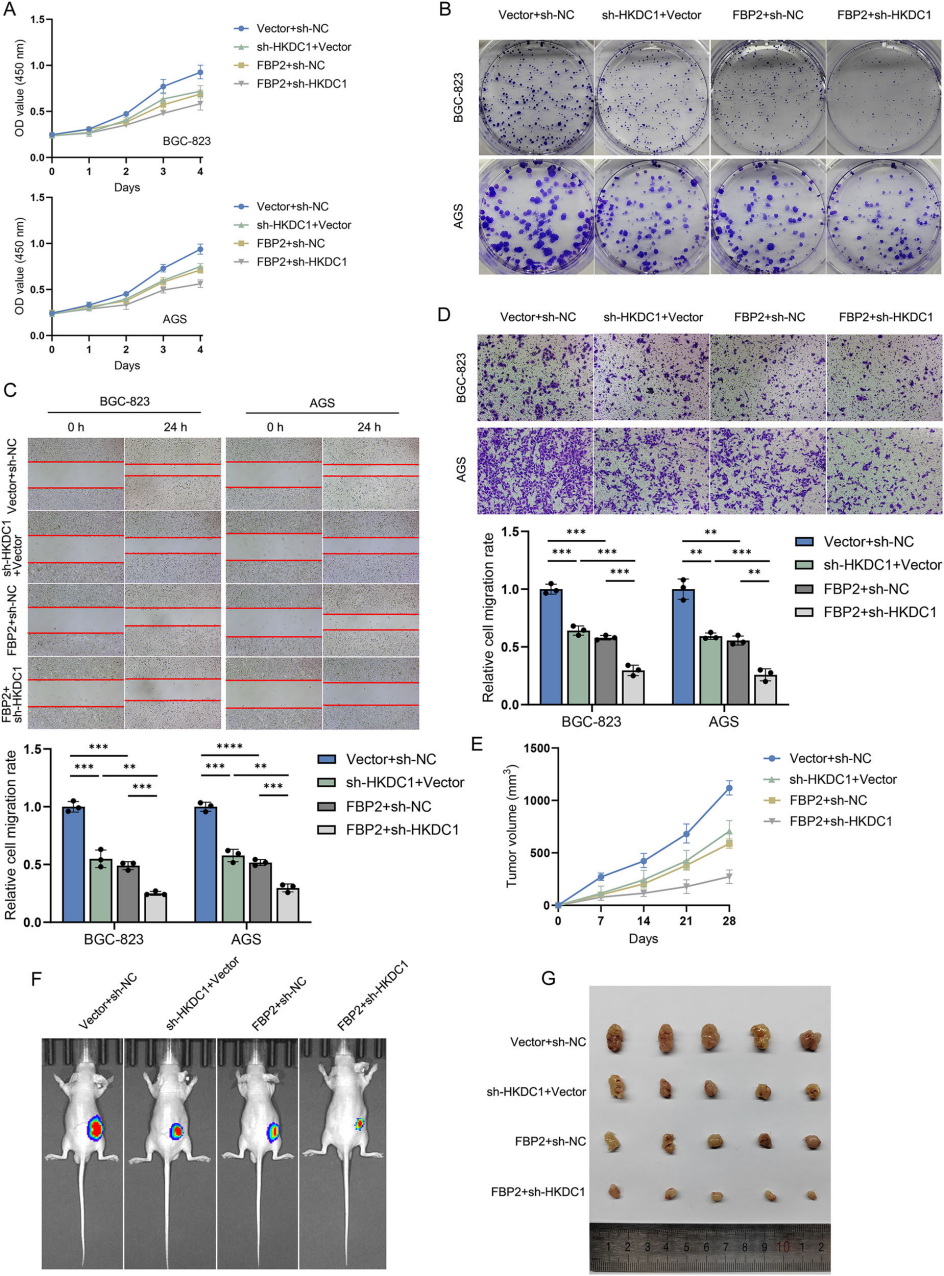
The synergistic inhibitory effect of FBP2 and sh-HKDC1 on the malignant biological behavior of GC cells in vitro and in vivo. **A** CCK-8 assays and **B** colony formation assays confirmed the synergistic inhibitory effect of FBP2+sh-HKDC1 on the proliferation of GC cells. **C** Scratch assays and **D** Transwell assays confirmed the synergistic inhibitory effects of FBP2+sh-HKDC1 on the migratory and invasive capabilities of GC cells. The bar chart presented the statistical analysis of the migration and invasion ratios. **E** Trend of subcutaneous tumor volume in nude mice following the injection of BGC-823 GC cells over a four-week period. **F** In vivo imaging and **G** tumor excision were evaluated in nude mice from the FBP2+sh-HKDC1, FBP2+sh-NC, sh-HKDC1+Vector, and Vector+sh-NC groups four weeks post-subcutaneous injection. **P < 0.01, ***P < 0.001, ****P < 0.0001.

A stable FBP2+sh-HKDC1 cell line was established through lentiviral transduction, which specifically knocks down HKDC1 expression in the FBP2 cell line. Subsequently, we investigated the malignant biological behaviors of FBP2+sh-HKDC1, FBP2+sh-NC, sh-HKDC1+Vector, and Vector+sh-NC cell lines through both in vitro and in vivo experiments. In vitro experiments, including CCK-8 (Fig. 8A), colony formation (Fig. 8B), cell scratch (Fig. 8C), and Transwell assays (Fig. 8D), demonstrated that FBP2+sh-HKDC1 exhibited the most significant inhibitory effects on the proliferation, migration, and invasion of GC cells. The results of subcutaneous tumor formation in nude mice were in agreement with the aforementioned in vivo experimental findings (Fig. 8E–G).

Subsequently, FBP2+sh-HKDC1 and Vector+sh-NC GC cells were collected for RNA sequencing analysis, and the DEGs with a Fold change ≥2 and *P* < 0.05 were identified.

**Fig. 9:**
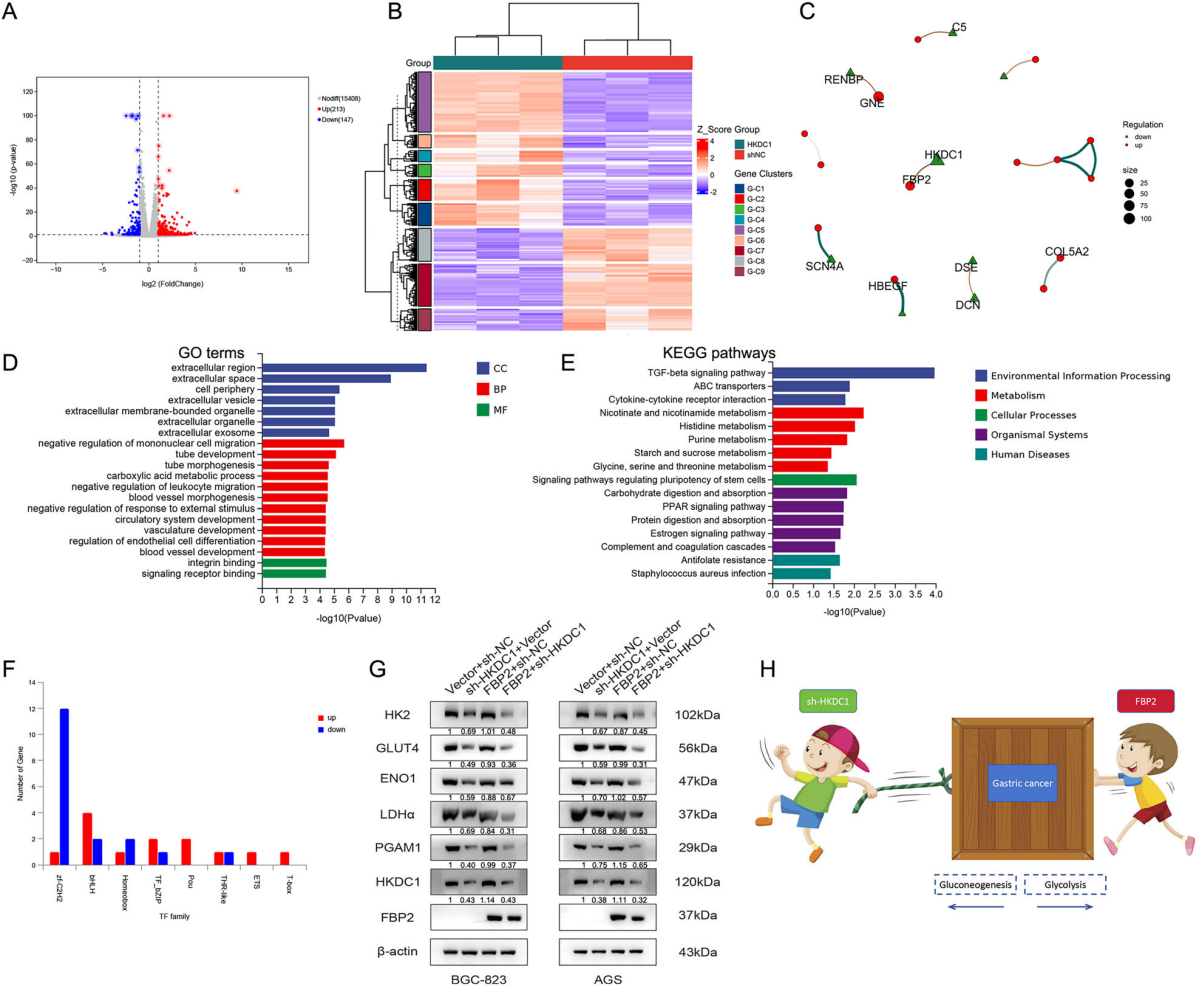
Analysis of the synergistic regulatory mechanism of FBP2+sh-HKDC1 on GC. **A** Volcano plot and **B** heatmap generated from the RNA sequencing data of FBP2+sh-HKDC1 and Vector+sh-NC cells, along with **C** PPI network constructed based on the top 10 significant DEGs. **D** GO and **E** KEGG pathway enrichment analysis was performed based on the DEGs. **F** Differentially expressed transcription factor families and their corresponding gene members. **G** Impact of FBP2+sh-HKDC1 on the expression levels of key glycolytic proteins in GC cells. **H** Schematic illustration of the synergistic regulatory mechanism of FBP2 and sh-HKDC1 on glucose metabolism in GC.

Volcano plot (Fig. 9A) and heatmap (Fig. 9B) were constructed based on DEGs. In addition, a PPI network was constructed based on the top 10 DEGs to illustrate their interaction relationships (Fig. 9C). Enrichment analyses were performed separately for the GO (Fig. 9D) and KEGG pathways (Fig. 9E) based on DEGs.

The analysis results indicated that environmental information processing, metabolism, and organismal systems were the primary categories of the enriched KEGG pathways. Notably, the transforming growth factor-β (TGF-β) signaling pathway played a crucial role. The differentially expressed transcription factor (TF) families and their corresponding gene members were presented in Fig. 9F. WB assay revealed that the expression levels of key glycolytic pathway proteins were markedly reduced in the FBP2+sh-HKDC1 group (Fig. 9G), indicating that the combined modulation of FBP2 and sh-HKDC1 can further diminish the aerobic glycolysis capacity of GC cells. A schematic diagram illustrating the synergistic effect of FBP2 and HKDC1 on tumor glucose metabolism was created (Fig. 9H).

## Discussion

For patients with locally advanced and metastatic GC, the current mainstream treatment strategy involves a multimodal approach combining surgery, chemoradiotherapy, targeted therapy, and immunotherapy. While this comprehensive treatment regimen has demonstrated benefits for patient survival, its overall efficacy remains limited [14, 15]. In recent years, targeted therapies, including Apatinib and Trastuzumab, have demonstrated promising efficacy in GC treatment. Nonetheless, the current therapeutic targets and targeted drugs for GC remain considerably limited, highlighting the urgent need to identify novel therapeutic targets [16, 17].

Gluconeogenesis is a metabolic pathway that converts non-carbohydrate precursors, including lactic acid, glycerol, and amino acids, into glucose. This process represents the reverse of glycolysis and primarily takes place in the liver and kidneys, with minor contributions from other tissues. In the majority of tumor tissues, gluconeogenesis is suppressed while aerobic glycolysis is significantly intensified. Thus, inhibiting aerobic glycolysis or enhancing the gluconeogenesis pathway holds potential as a strategy to impede tumor progression [18, 19]. FBP1, serving as a critical rate-limiting enzyme in gluconeogenesis, has been documented to play a significant role in various tumors. However, there has been limited research exploring the relationship between FBP2 and cancer [20, 21]. Studies conducted by Duda et al. demonstrated that healthy fibroblasts establish a metabolic symbiosis with non-small cell lung cancer cells. This is achieved through the down-regulation of glycolytic enzyme expression in cancer cells and an enhanced capacity of fibroblasts to release lactic acid, consequently supplying the cancer cells with energy-rich glucose-derived metabolites. Further experiments demonstrated that the aforementioned alterations were attributable to elevated FBPase expression, which subsequently modulated HIF-1α function in fibroblast-stimulated cancer cells. In lung cancer cells, the predominant protein that exhibits a strong interaction with HIF-1α is FBP2, rather than FBP1 [22].

As a pivotal transcriptional regulator of the adaptive response to hypoxia, HIF-1α can activate the transcription of over 40 genes under hypoxic conditions. These genes include those encoding erythropoietin, glucose transporters, glycolytic enzymes, vascular endothelial growth factor, and other protein products that enhance oxygen delivery or promote metabolic adaptation to hypoxia. HIF-1α plays a crucial role in tumor angiogenesis and the pathophysiology of ischemic diseases [23–25]. The existing literature has confirmed that HIF-1α facilitates the adaptation of tumor cells to hypoxic environments through mechanisms such as inducing neovascularization, thereby improving the hypoxic conditions and promoting tumor proliferation, invasion, and metastasis. This has been proven to play a crucial role in various tumors, including GC [26, 27]. To date, there has been no reported research on the interaction and regulatory mechanisms between HIF-1α and FBPase in GC [28]. In this study, we elucidated the interaction between HIF-1α and FBP2 in GC for the first time using immunofluorescence double labeling, CO-IP, and molecular docking experiments. Furthermore, the results from qRT-PCR and WB experiments confirmed that FBP2 leads to reduced levels of HIF-1α in GC cells.

The results of RNA sequencing and glycolysis assays demonstrated that FBP2 can enhance oxidative phosphorylation while attenuating aerobic glycolysis in GC cells. Therefore, the mechanism by which FBP2 inhibits the malignant biological behavior of GC is multifaceted and complex. On one hand, FBP2 can indirectly attenuate the glycolysis process by promoting gluconeogenesis. On the other hand, FBP2 can ameliorate the hypoxic microenvironment by downregulating the expression level of HIF-1α, thereby enhancing oxidative phosphorylation in GC cells and attenuating their aerobic glycolysis process [29, 30]. This study identified and validated the interaction between FBP2 and HIF-1α in GC for the first time, elucidating the mechanism by which FBP2 suppresses the malignant progression of GC through the regulation of HIF-1α and inhibition of glucose metabolic reprogramming. HIF-1α appears as a key mediator, yet its regulation by FBP2 is only partially characterized. Is the interaction direct or indirect? What domains are involved? Does FBP2 affect HIF-1α stability or transcriptional activity? The answers to these questions remain unknown at present. This opens a promising line of inquiry that remains underdeveloped. The oligomerization state and functional regulation of FBP2 are modulated by AMP and NAD+, which serve as critical indicators of cellular metabolic status. Therefore, it can be hypothesized that the metabolic regulation mediated by HIF-1α in cancer is modulated by FBP2, which functions as a sensor to detect and relay signals related to cellular energy and REDOX status [22].

Huangyang et al. discovered that FBP2 expression is silenced across multiple subtypes of soft tissue sarcoma, and they demonstrated that the enforced expression of FBP2 inhibits both sarcoma cell proliferation and tumor growth via two distinct mechanisms. First, FBP2 in the cytoplasm counteracts the elevated glycolysis associated with the Warburg effect, thereby suppressing sarcoma cell proliferation. Secondly, the nuclear localization of FBP2 can suppress the expression of the mitochondrial transcription factor A (TFAM), which is dependent on c-Myc, thereby inhibiting mitochondrial biogenesis and respiration [31]. Wang et al. reported a significantly reduced FBP2 expression in cervical cancer tissues, which was correlated with shorter OS. FBP2 may inhibit aerobic glycolysis by promoting the ubiquitination of pyruvate kinase isoenzyme M2 (PKM2), thereby impeding the proliferation of cervical cancer cells [32]. In this study, we did not observe a significant impact of FBP2 on the expression levels of c-Myc in GC cells. Despite the ability of FBP2 to decrease the expression levels of HIF-1α and GPI in GC cells, it concurrently increases the expression level of HKDC1. This upregulation of HKDC1 may partially mitigate the tumor-inhibitory effects of FBP2 [33, 34].

HKDC1 represents the fifth recently identified member of the hexokinase family. Given its structural similarity to other hexokinases, HKDC1 is hypothesized to play a comparable role in tumor progression through the modulation of aerobic glycolysis. To date, HKDC1 has been identified as being highly expressed in endometrial, gastric, and hepatic carcinomas, potentially playing a significant role in the onset, progression, and prognosis of various malignant solid tumors [35, 36]. The study conducted by Fang et al. demonstrated a significant correlation between HKDC1 and GC induced by Helicobacter pylori. In vitro and in vivo studies have demonstrated that Helicobacter pylori infection upregulates TGF-β1 and p-Smad2, consequently activating the epithelial-mesenchymal transition (EMT) pathway, wherein HKDC1 plays a pivotal role [37]. Wang et al. found that the upregulation of HKDC1 can enhance glycolysis, cell proliferation, and tumorigenesis. HKDC1 facilitates the invasion and metastasis of GC through the induction of EMT [34]. In accordance with prior research findings, our study also demonstrated a reduced glycolysis rate of sh-HKDC1 GC cells, along with an attenuated malignant biological behavior. In the WB assays of key glycolytic proteins, we measured the expression levels of HK2. The WB results demonstrated that the expression levels of HK2 protein were reduced to varying extents in both the sh-HKDC1 and FBP2+sh-HKDC1 groups. Therefore, the potential compensatory effects of other hexokinase family members for HKDC1 loss were excluded.

Another key finding of this study is that FBP2 can positively regulate the expression of HKDC1. The specific action mechanism underlying the aforementioned regulation is intricate, and both compensatory and context-dependent mechanisms may be involved. The impact of HKDC1 on tumor glucose metabolism differs significantly from that of HK2 [38]. Glycolysis assays indicated that FBP2 results in a reduction in HK2 expression, whereas HKDC1 may partially compensate for the loss of HK2 function through increased expression. According to prior research, HKDC1 plays a crucial role in lipid metabolism, the tricarboxylic acid (TCA) cycle, and mitochondrial function of tumor cells [33, 38]. FBP2, which is distributed in both the cytoplasm and nucleus, inhibits tumor growth by antagonizing glycolysis and mitochondrial function, respectively, thereby aligning with the action pathway of HKDC1. Consequently, FBP2 may redirect the energy metabolism of tumor cells towards lipid metabolism through the interplay of glycolysis and lipid metabolism, and can also influence the TCA cycle and mitochondrial function by ameliorating the hypoxic microenvironment [31]. As a pivotal participant in the aforementioned reactions, the expression and function of HKDC1 are also upregulated correspondingly. The synergistic effect of FBP2 and sh-HKDC1 further suppresses aerobic glycolysis in GC cells, thereby enhancing the anti-tumor efficacy. Our study focused on verifying the interaction between FBP2 and HIF-1α, while also conducting a preliminary exploration of the synergistic effects of FBP2 and HKDC1. Notably, this research is the first to identify and elucidate the interaction between FBP2 and HKDC1 in GC, as well as their synergistic anti-tumor effects. Prior to exploring the combined regulation of FBP2+sh-HKDC1, we had individually validated the effects of single-target interventions targeting FBP2 and HKDC1. Therefore, in this study, we did not reintroduce HKDC1 to rescue the tumor-suppressive phenotype associated with FBP2+sh-HKDC1. In the subsequent research, we will further investigate and validate the interaction and regulatory mechanisms between FBP2 and HKDC1.

We anticipate utilizing FBP2 as a bridging molecule to cross-link HIF-1α and HKDC1, which are regulated by FBP2, with metabolic reprogramming and the progression of malignant biological behaviors in GC. This study elucidated the critical role of the FBP2/HIF-1α signaling axis and the synergistic regulation of FBP2 and HKDC1 in governing the occurrence, progression, and fate of GC cells, thereby providing robust evidence to support further investigation into the clinical translational potential of FBP2 inhibitors and dual-target therapies. According to prior research, the shift in tumor energy supply from glucose metabolism to lipid metabolism represents a critical factor that constrains both the clinical applicability and efficacy of ketogenic diet therapy [39, 40]. In light of the pivotal roles of FBP2 and HKDC1 in glycolipid metabolism and the TCA cycle, the dual-target intervention involving FBP2+sh-HKDC1 in combination with a ketogenic diet is anticipated to further enhance the anti-tumor efficacy and improve patient prognosis.

The present study still possesses certain limitations. Firstly, this study identified the target of FBP2 through bioinformatics analysis and previous literature, followed by experimental validation. However, it is important to acknowledge that other interacting proteins may also play a role in the regulation of FBP2. Secondly, the immunofluorescence co-localization experiments and molecular docking analyses indicated a potential direct interaction between FBP2 and HIF-1α. To further investigate the dynamic changes of proteins within cells, CO-IP and WB analyses were performed. However, additional validations, such as truncated mutation experiments of FBP2 or HIF-1α, were not performed, thus failing to provide further evidence for the interaction. Thirdly, this study examined the synergistic inhibitory effect of FBP2 and HKDC1 on the malignant biological behavior of GC cells. However, an in-depth investigation into the interaction between FBP2 and HKDC1 was not conducted.

## Conclusion

Low expression of FBP2 was associated with adverse clinicopathological characteristics and unfavorable prognosis in patients with GC. Overexpression of FBP2 in GC cells resulted in a reduced expression level of HIF-1α, enhanced oxidative phosphorylation, and a modest decrease in glycolytic activity, thereby exerting an anti-tumor effect. Notably, the overexpression of FBP2 has been shown to elevate the expression level of HKDC1. The synergistic effect of FBP2 and sh-HKDC1 further suppressed GC progression by promoting oxidative phosphorylation and inhibiting glycolysis, thereby enhancing the anti-tumor efficacy. This study elucidated the critical role of the FBP2/HIF-1α signaling axis and the synergistic regulation of FBP2 and HKDC1 in influencing the initiation, progression, and prognosis of GC. It provides substantial evidence to support further investigation into the clinical translational potential of FBP2 inhibitors and dual-target therapies.

## Supplementary information

Original WB data

Supplementary Material 1

Supplementary Material 2

Supplementary Material 3

## Data Availability

The data supporting the findings of this study are available from the corresponding author upon reasonable request.
