# Supplementary material for: The regulatory and synergistic effects of FBP2 and HKDC1 on glucose metabolism and malignant progression in gastric cancer
Source: Cell Death Dis. 2025 Oct 16;16(1):730. doi: 10.1038/s41419-025-07997-z (PMC12533130; doi:10.1038/s41419-025-07997-z)
Supplement: Supplementary file 1 — Original WB data [file 41419_2025_7997_MOESM1_ESM.pdf]

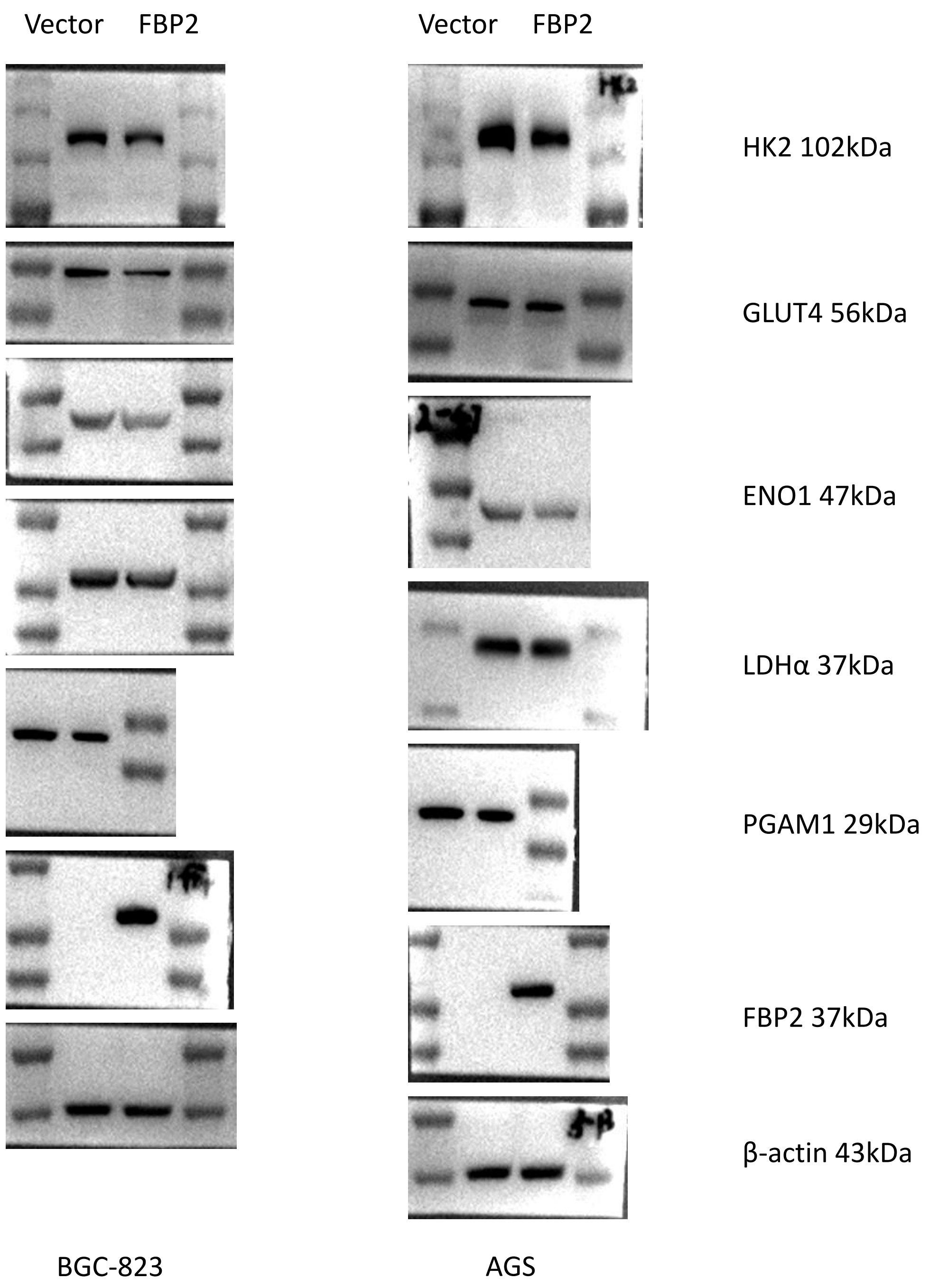
Vector
FBP2
Vector
FBP2
HK2 102kDa
GLUT4 56kDa
ENO1 47kDa
LDHα 37kDa
PGAM1 29kDa
FBP2 37kDa
β-actin 43kDa
BGC-823
AGS

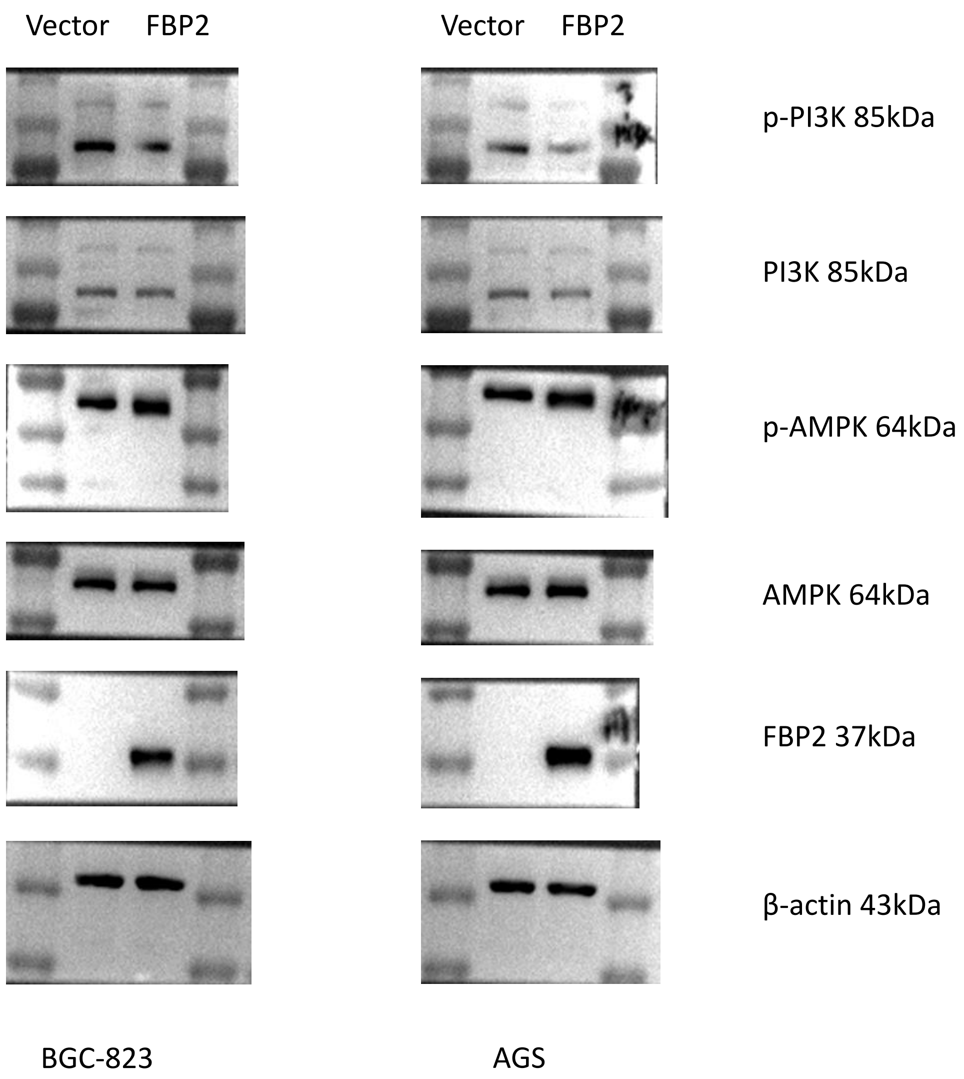
Vector
FBP2
Vector
FBP2
p-PI3K 85kDa
PI3K 85kDa
p-AMPK 64kDa
AMPK 64kDa
FBP2 37kDa
β-actin 43kDa
BGC-823
AGS

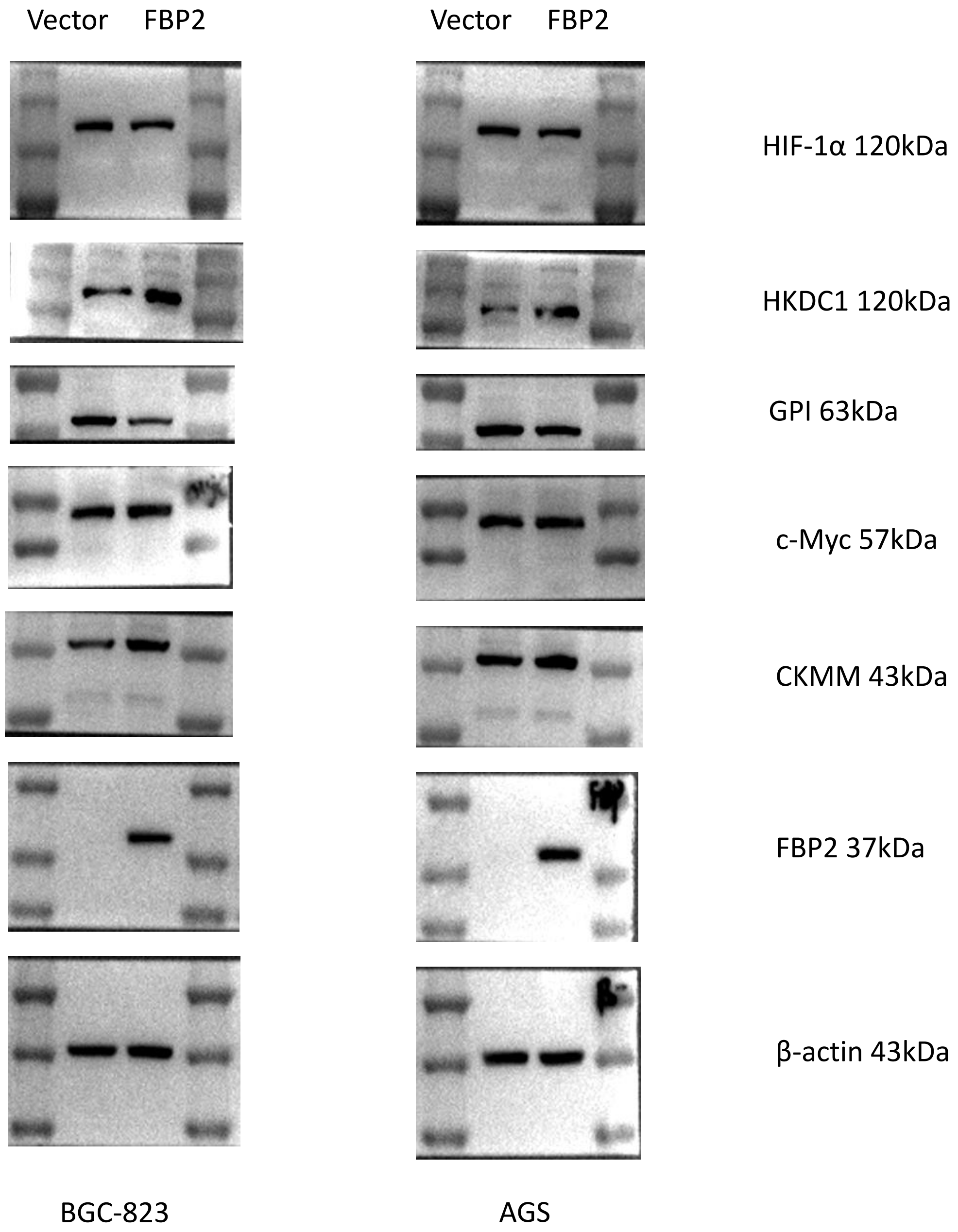
Vector
FBP2
Vector
FBP2
HIF-1α 120kDa
HKDC1 120kDa
GPI 63kDa
c-Myc 57kDa
CKMM 43kDa
FBP2 37kDa
β-actin 43kDa
BGC-823
AGS

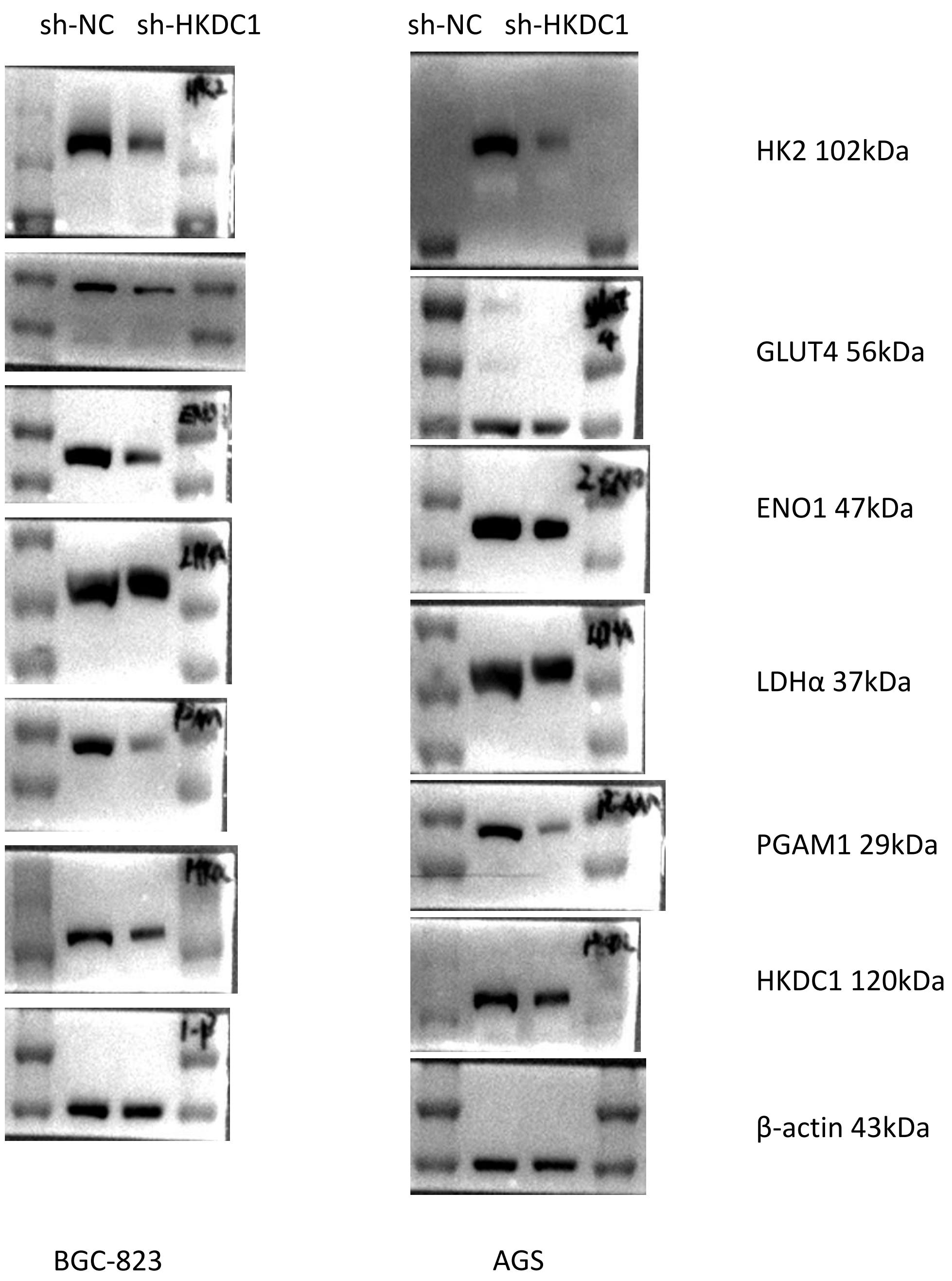

sh-NC
sh-HKDC1
sh-NC
sh-HKDC1
HK2 102kDa
GLUT4 56kDa
ENO1 47kDa
LDHα 37kDa
PGAM1 29kDa
HKDC1 120kDa
β-actin 43kDa
BGC-823
AGS

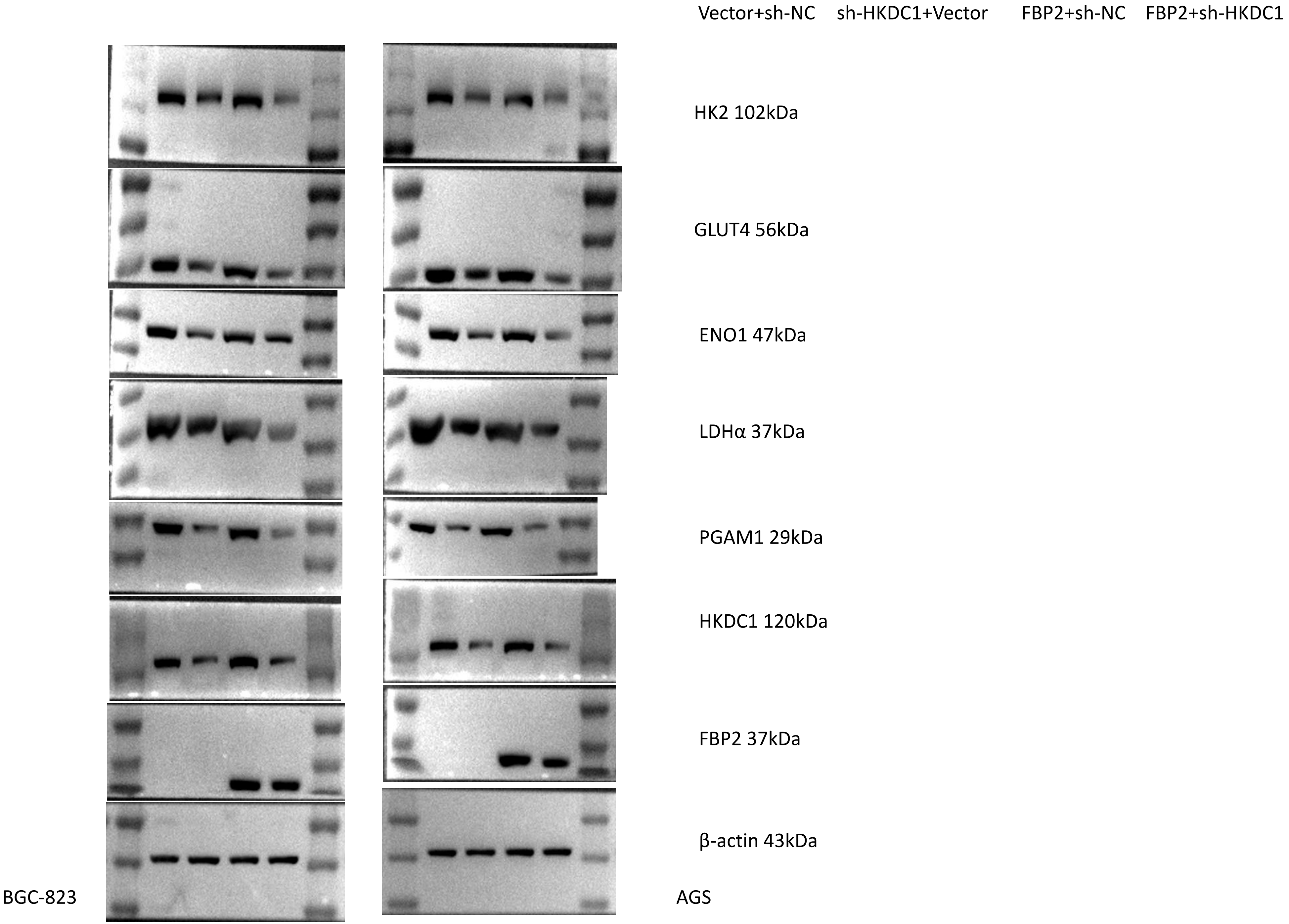
Vector+sh-NC
sh-HKDC1+Vector
FBP2+sh-NC
FBP2+sh-HKDC1
HK2 102kDa
GLUT4 56kDa
ENO1 47kDa
LDHα 37kDa
PGAM1 29kDa
HKDC1 120kDa
FBP2 37kDa
β-actin 43kDa
BGC-823
AGS
