# Supplementary material for: The regulatory and synergistic effects of FBP2 and HKDC1 on glucose metabolism and malignant progression in gastric cancer
Source: Cell Death Dis. 2025 Oct 16;16(1):730. doi: 10.1038/s41419-025-07997-z (PMC12533130; doi:10.1038/s41419-025-07997-z)

**Supplementary material 1** The nucleotide sequences and plasmids used in this study.

| Definition | Sequence(5’-3’) |
| --- | --- |
| sh-HKDC1 | GTTGCAGTCGTGAATGATACA |
| sh-NC | GCTCGCCTGTCTACTAACTAA |


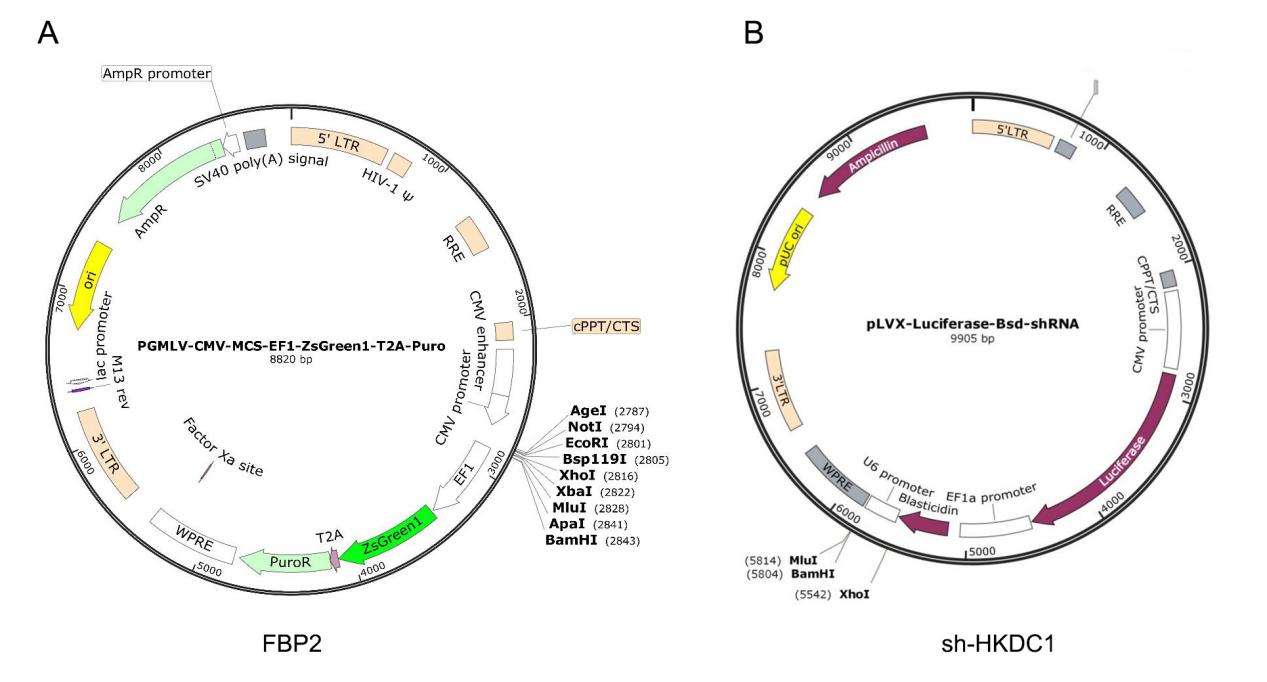

Supplement: Supplementary file 2 — Supplementary Material 1 [file 41419_2025_7997_MOESM2_ESM.docx]
