# Supplementary material for: The regulatory and synergistic effects of FBP2 and HKDC1 on glucose metabolism and malignant progression in gastric cancer
Source: Cell Death Dis. 2025 Oct 16;16(1):730. doi: 10.1038/s41419-025-07997-z (PMC12533130; doi:10.1038/s41419-025-07997-z)
Supplement: Supplementary file 3 — Supplementary Material 2 [file 41419_2025_7997_MOESM3_ESM.docx]

**Supplementary material 2** The primers used in this study.

| Name | Primer | Sequence |
| --- | --- | --- |
| FBP2 | Forward primer | ACCCGCTACGTTATGGAAAAG |
|  | Reverse primer | GCCGTCAGCATTGAGTTCAG |
| HKDC1 | Forward primer | GAAGGTGCAAGTCGCTGAAGA |
|  | Reverse primer | GTCCACGAAAGTAGGACACCC |
| CKM | Forward primer | CTGACAAGCACAAGACTGACC |
|  | Reverse primer | CTGCTGAGCACGTAGTTAGGG |
| GPI | Forward primer | CAAGGACCGCTTCAACCACTT |
|  | Reverse primer | CCAGGATGGGTGTGTTTGACC |
| HIF-1α | Forward primer | GAACGTCGAAAAGAAAAGTCTCG |
|  | Reverse primer | CCTTATCAAGATGCGAACTCACA |
| c-Myc | Forward primer | GTCAAGAGGCGAACACACAAC |
|  | Reverse primer | TTGGACGGACAGGATGTATGC |
| GAPDH | Forward primer | ACCCACTCCTCCACCTTTGAC |
|  | Reverse primer | TGTTGCTGTAGCCAAATTCGT |
