# Supplementary material for: The regulatory and synergistic effects of FBP2 and HKDC1 on glucose metabolism and malignant progression in gastric cancer
Source: Cell Death Dis. 2025 Oct 16;16(1):730. doi: 10.1038/s41419-025-07997-z (PMC12533130; doi:10.1038/s41419-025-07997-z)
Supplement: Supplementary file 4 — Supplementary Material 3 [file 41419_2025_7997_MOESM4_ESM.docx]

**Supplementary material 3** The antibodies used in this study.

| Antibodies | Manufacturers | Applications |
| --- | --- | --- |
| FBP2 | Abcam, ab131253 | 1:1000 for WB, 1:100 for IHC |
| FBP2 | Santa, sc-271799 | 1:1000 for CO-IP, 1:50 for IF |
| HKDC1 | ABclonal, A16573 | 1:1000 for WB |
| HIF-1α | Abcam, ab308433 | 1:1000 for WB and CO-IP,  1:100 for IF |
| HK2 | CST, 2867S | 1:1000 for WB |
| GLUT4 | Abcam, ab35826 | 1:1000 for WB |
| ENO1 | CST, 3810S | 1:1000 for WB |
| LDHα | CST, 3582S | 1:1000 for WB |
| PGAM1 | Abcam, ab288376 | 1:1000 for WB |
| AMPK | Abcam, ab32047 | 1:1000 for WB |
| phospho-AMPK | Abcam, ab92701 | 1:1000 for WB |
| PI3K | ABclonal, A4992 | 1:1000 for WB |
| phospho-PI3K | ABclonal, AP0427 | 1:1000 for WB |
| GPI | Servicebio, GB113691 | 1:1000 for WB |
| c-Myc | Abcam, ab185655 | 1:1000 for WB |
| CKMM | Servicebio, GB113064 | 1:1000 for WB |
| β-actin | Servicebio, GB15003-100 | 1:1000 for WB |
